# The Transcriptional Factor PPARαb Positively Regulates *Elovl5* Elongase in Golden Pompano *Trachinotus ovatus* (Linnaeus 1758)

**DOI:** 10.3389/fphys.2018.01340

**Published:** 2018-09-25

**Authors:** Ke-Cheng Zhu, Ling Song, Chao-Ping Zhao, Hua-Yang Guo, Nan Zhang, Liang Guo, Bao-Suo Liu, Shi-Gui Jiang, Dian-Chang Zhang

**Affiliations:** ^1^Key Laboratory of South China Sea Fishery Resources Exploitation and Utilization, Ministry of Agriculture and Rural Affairs – South China Sea Fisheries Research Institute, Chinese Academy of Fishery Sciences, Guangzhou, China; ^2^Guangdong Provincial Engineer Technology Research Center of Marine Biological Seed Industry, Guangzhou, China; ^3^Guangdong Provincial Key Laboratory of Fishery Ecology and Environment, Guangzhou, China; ^4^College of Fisheries and Life Science, Shanghai Ocean University, Shanghai, China

**Keywords:** *Trachinotus ovatus*, promoter activity, transcription factors, PPARα, Elovl5

## Abstract

The nuclear peroxisome proliferator-activated receptors (PPARs) regulate the transcription of elongases of very long-chain fatty acids (Elovl), which are involved in polyunsaturated fatty acid (PUFA) biosynthesis in mammals. In the present study, we first characterized the function of *Elovl5* elongase in *Trachinotus ovatus*. The functional study showed that *ToElovl5* displayed high elongation activity toward C18 and C20 PUFA. To investigate whether PPARαb was a regulator of *Elovl5*, we also reported the sequence of *T. ovatus PPARαb* (*ToPPARαb*). The open reading frame (ORF) sequence encoded 469 amino acids possessing four typical characteristic domains, including an N-terminal hypervariable region, a DNA-binding domain (DBD), a flexible hinge domain and a ligand-binding domain (LBD). Thirdly, promoter activity experiments showed that the region from PGL3-basic-Elovl5-5 (-146 bp to +459 bp) was defined as the core promoter by progressive deletion mutation of *Elovl5*. Moreover, PPARαb overexpression led to a clear time-dependent enhancement of *ToElovl5* promoter expression in HEK 293T cells. Fourth, the agonist of PPARαb prominently increased *PPARαb* and *Elovl5* expression, while PPARαb depletion by RNAi or an inhibitor was correlated with a significant reduction of *Elovl5* transcription in *T. ovatus* caudal fin cells (TOCF). In conclusion, the present study provides the first evidence of the positive regulation of *Elovl5* transcription by PPARαb and contributes to a better understanding of the transcriptional mechanism of PPARαb in fish.

## Introduction

Long-chain polyunsaturated fatty acids (LC-PUFA) biosynthesis initiates from C_18_ PUFA and requires a series of elongation and desaturation steps catalyzed by elongases of very long chain fatty acids (Elovls) and fatty acid desaturases (Fads) in vertebrates (Cook and McMaster, 2004; Castro et al., 2016). PPARα is a ligand-activated nuclear transcription factor from the steroid receptor superfamily that regulates LC-PUFA biosynthesis (Kota et al., 2005; Sampath and Ntambi, 2005). In mammals, PPARα is activated by fatty acids or their derivatives and plays pleiotropic roles in lipid metabolism, such as stimulating the expression of genes related to peroxisomal and mitochondrial fatty acid oxidation and LC-PUFA biosynthesis (Desvergne et al., 2006). PPARα agonists (WY14643) affect fatty acid elongation pathways, thereby increasing *Elovl5* expression in adult *Rattus norvegicus* (Wang et al., 2005). Moreover, in PPARα-defective mice, PPARα was required for the WY14643-mediated induction of *Elovl5* and *Elovl6* (Wang et al., 2006). Cold-induced *Elovl3* mRNA levels were under the control of PPARα in *Mus musculus* (Jakobsson et al., 2005). Nevertheless, the role of PPARα in the expression of *Elovl5* is less understood in fish. Furthermore, PPARα stimulates the expression of target genes directly through binding to PPAR response elements (PPREs) in the promoter regions of target genes. Dong et al. (2017) indicated that PPARα bound to the *Fads2* promoter region and upregulated the transcription of *Fads2* in fish. PPARα has been implicated as a *trans*-acting factor that promotes insulin-induced gene (Insig2a) expression, consequently suppressing sterol-regulatory element binding protein 1c (SREBP-1c) processing during fasting (Lee et al., 2017).

The rate-limiting condensation step is catalyzed by Elovls in the elongation of fatty acids in LC-PUFA biosynthesis (Nugteren, 1965; Jakobsson et al., 2006). *Elovl5* has been verified and functionally characterized as a critical enzyme in the elongation step of LC-PUFA biosynthesis (Castro et al., 2016; Li et al., 2017; Lin et al., 2018). *Elovl5* could effectively elongate C18, C20, and C22 PUFAs and has been isolated from various teleost species (Bell and Tocher, 2009; Monroig et al., 2012; Xie et al., 2016). In fish, *Elovl5* was isolated, and in PUFA biosynthesis, it was consistent with that in mammals and invertebrates (Monroig et al., 2012; Gregory and James, 2014; Kabeya et al., 2015; Li et al., 2016), suggesting a conserved function of *Elovl5* in metazoans.

Teleost fish, particularly marine fish, are unique and rich sources of omega-3 (n-3) LC-PUFAs in the human diet (Tocher, 2015). The golden pompano *Trachinotus ovatus* (Linnaeus 1758), Carangidae, and Perciformes are broadly cultivated in the Asia-Pacific region and considered important aquaculture species in China (Sun et al., 2014; Zhen et al., 2014). Furthermore, high levels of LC-PUFA content were detected in *T. ovatus* muscle (Zhang et al., 2010). Hence, to investigate whether *T. ovatus* PPARαb (ToPPARαb) would be a mediator of *ToElovl5*, the sequence characterization, tissue distribution and transcriptional regulation of *ToPPARαb* were determined. The present study of ToPPARαb presents a potential molecular pathway of LC-PUFA biosynthesis mechanisms.

## Materials and Methodss

### Ethics Statement

All experiments in this study were approved by the Animal Care and Use Committee of South China Sea fisheries Research Institute, Chinese Academy of fishery Sciences (No. SCSFRI96-253) and performed according to the regulations and guidelines established by this committee.

### Gene Cloning and Bioinformatics

The *Elovl5* and *PPARαb* predicted sequence were obtained from genomic data for *T. ovatus* (Accession No. PRJEB22654 under ENA, Sequence Read Archive under BioProject PRJNA406847). To determine the accuracy of the encoding sequence of *Elovl5* and *PPARαb*, gene-specific primers were designed (**Supplementary Table S1**) based on the putative sequence. Total RNA (1 μg) was extracted from *T. ovatus* liver (Trizol reagent, Invitrogen, United States) and was reverse transcribed into cDNA by random hexamer primers (Cloned AMV First-Strand cDNA Synthesis Kit, Invitrogen, United States). The 3′ of the transcript was cloned from liver cDNA using specific primers with the SMART^TM^ RACE cDNA amplification kit (Clontech, Mountain View, CA, United States). PCR was conducted as previously described (Zhu et al., 2014).

Amino acid sequence of ToPPARαb was used as queries to search for the homologous genes in NCBI database^1^. All available PPARα genes and mature peptides were downloaded from Ensembl^2^ and Genome Browser^3^. The gene structure was predicted by the SANTA CRUZ Genome Browser (see footnote 3), and signal peptides were detected with SignalP software^4^. Molecular weight and theoretical isoelectric point were calculated by Compute pI/Mw software^5^. A three-dimensional (3D) model of the ToPPARαb amino acid sequence was developed by the SWISS-MODEL Protein Modelling Server. To better understand the relationship of PPARαs in metazoans, all PPARα amino acid sequences were aligned by ClustalW2^6^. Artificially arranged the ambiguously aligned sequences, and then a maximum likelihood (ML) phylogenetic tree (LG + G model, bootstrap 1000) of PPARα putative proteins was constructed by MEGA 6 software (Tamura et al., 2013).

### Heterologous Expression of the *ToElovl5* Elongase ORFs in Yeast

PCR fragment corresponding to the ORF of the *Elovl5* elongase was amplified from *T. ovatus* liver cDNA using primers that included *Hind*III and *Xho*I restriction sites (**Supplementary Table S1**). Subsequently, the DNA fragment was digested with the relevant restriction endonucleases (New England BioLabs, Herts, United Kingdom) and ligated into a coincident restricted pYES2 yeast expression vector (Invitrogen, Paisley, United Kingdom). The recombinant plasmid (pYES2-Elovl5) was then used to transform *Saccharomyces cerevisiae* competent cells (S.c. EasyComp Transformation Kit, Invitrogen). Transformation and selection of yeast with recombinant plasmids, and yeast culture were prepared according to previously described methods (Li et al., 2017). Fatty acids are: 18:3n-3 (α-linolenic acid), 18:3n-6 (γ-linolenic acid), 18:4n-3 (stearidonic acid), 20:4n-6 (arachidonic acid, ARA) and 20:5n-3 (eicosapentaenoic acid, EPA) were used as substrates for detecting the elongase activity of *ToElovl5*. Final concentrations of FA substrates varied according to their fatty acyl chain lengths, 0.5 mM (C18) and 0.75 mM (C20). Yeast cultures were incubated for 2 days at 30°C, and then were harvested, washed twice as described previously (Li et al., 2010). Under the same conditions, yeast transformed with pYES2 contain no insert was grown as a control.

### Plasmid Construction, Cell Culture, and Dual-Luciferase Reporter Assays

Total DNA was extracted from *T. ovatus* muscle using a Genomic DNA Isolation Kit (Invitrogen, United States). To investigate the role of PPARαb in the transcriptional regulation of *ToElovl5*, five different promoter regions of *ToElovl5* were amplified by specific primers (**Supplementary Table S1**) and subcloned into the *Kpn*I and *Xho*I restriction sites of the pGL3-basic luciferase reporter plasmid (Promega, United States). Five recombinant plasmids, denoted pGL3-basic-Elovl5-1 (-382 to +89), pGL3-basic-Elovl5-2 (-793 to +89), pGL3-basic-Elovl5-3 (-1262 to +89), pGL3-basic-Elovl5-4 (-146 to +265) and pGL3-basic-Elovl5-5 (-146 to +459), were constructed (**Figure 5**). Moreover, the ORF of *ToPPARαb* was amplified with primers including restriction sites for *Nhe*I and *Hind*III, respectively. The DNA fragment was digested with the corresponding restriction endonucleases (Takara, Japan) and ligated into a pCDNA3.1 vector (Invitrogen, United States).

The Renilla luciferase plasmid pRL-TK (Promega, United States) was used as an internal control. Plasmids for transfection were prepared using the TransGen Plasmid Mini Kit (Beijing, China). Human embryonic kidney (HEK 293T) and *T. ovatus* caudal fin (TOCF) cell culture and transfection experiments were performed according to Li et al. (2017) and Wei et al. (2018), respectively.

### *PPARαb* Overexpression and Knockdown

RNA interference (siRNA) of PPARαb (PPARαb-si) and corresponding negative controls (si-NC) were purchased from Genecreate (Wuhan, China). Lipofectamine RNAiMAX transfection reagent (Invitrogen, United States) was used for transfection in TOCF cells. The PPARαb siRNA sequence is listed in **Supplementary Table S1**. Additionally, the agonist and inhibitor of PPARα were used to clarify the role of the transcription factor in the regulation of *ToElovl5* elongases. WY-14643 (0.1, 1, and 4 μmol/L, Sigma, United States) was used as a PPARαb agonist, whereas GW6471 (0.1, 1, and 4 μmol/L, Sigma, United States) was used as a PPARαb inhibitor. Total RNA was extracted from TOCF cells as described above. The experiment was performed according to Li et al. (2017).

### Quantitative Real-Time PCR

The tissue distributions of *PPARαb* mRNA levels were described by quantitative real-time polymerase chain reaction (qRT-PCR) using adult *T. ovatus* tissues (*n* = 6), including small intestine, liver, white muscle, brain, spleen, fin, gill, head-kidney, stomach, blood, and male (*n* = 3) and female gonad (*n* = 3) cDNA, as templates. Then, total RNA was isolated from 12 tissues as described above. The PrimeScript^®^ RT reagent Kit with gDNA Eraser (Takara, Japan) was used to synthesize cDNA from total RNA (1 μg). Specific primers and the housekeeping gene *EF-1α* (elongation factor 1, alpha) are displayed in **Supplementary Table S1**. The qRT-PCR was performed as previously described (Zhang et al., 2018). Relative expression was evaluated by the 2^-ΔΔC_T_^ method (Livak and Schmittgen, 2001).

### Statistical Analysis

Statistical analysis was performed using SPSS 19.0 software (IBM, United States). The data from different tissues and groups were analyzed by the Duncan test using one-way ANOVA. Data are shown as the means ± SD, and *p* < 0.05 indicates statistical significance.

## Results

### Sequence Characterization of ToElovl5 and ToPPARαb

**FIGURE 1:**
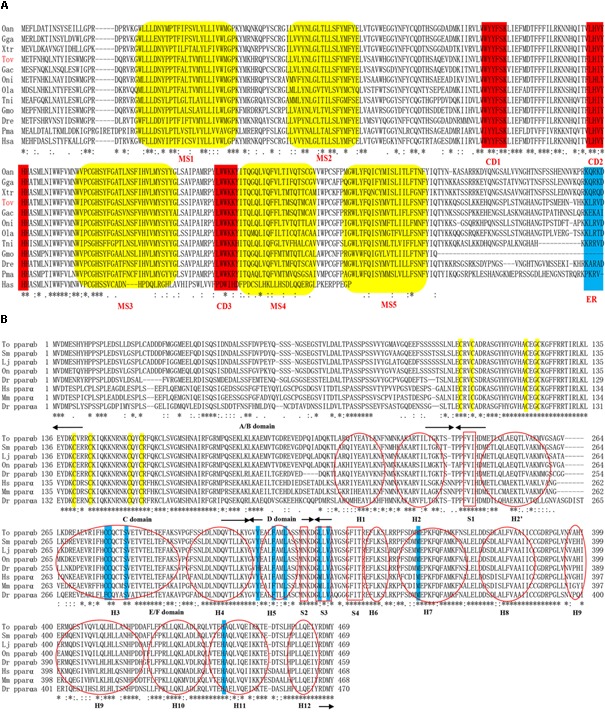
Amino acid sequences of Elovl5 **(A)** and PPARαb **(B)** homologs in vertebrate. **(A)** Indicated are the highly conserved domains (CD1-3), five putative membrane-spanning domains (MS1-5) and the ER retrieval signal. **(B)** The four domains indicated by arrows are the N-terminal hypervariable region (A/B), DNA-binding domain (C), flexible hinge domain (D), and ligand-binding domain (E/F). Yellow and blue outlines indicate the eight zinc-binding sites in the DBD and the nine ligand-binding sites in the LBD, respectively. Moreover, the 12 α-helices (H) and four parts of the β-sheet (S) are indicated by a red oval and box, respectively. The accession numbers of the Elovl5 and PPARαb sequences used and species abbreviation are listed in **Supplementary Table S3**.

The genomic sequence of *ToElovl5* elongase is 6,617 bp, including seven exons and six introns, while the full-length cDNA sequence is 3,764 bp, containing 185 bp of 5′ untranslated region (5′-UTR), a 885 bp ORF encoding a polypeptide of 294 amino acids and a 2,694 bp 3′-UTR including a polyA signal sequence (GenBank accession number: KY860144; **Supplementary Figure S1**). Furthermore, similar to other teleost Elovl5 proteins, *ToElovl5* deduced proteins possess three highly conserved domains (CD1-3), including the histidine box motif (HXXHH) (CD2), conserved in the elongase family (**Figure 1A**) (Xie et al., 2016). KXRXX motif was regarded as putative endoplasmic reticulum (ER) retention signal in Elovl5 carboxyl terminal (C-terminal). Five putative transmembrane-spanning regions, including hydrophobic amino acid (aa) stretches were predicted by comparison with other vertebrate Elovl proteins.

The genomic sequence of *ToPPARαb* is a 13,262 bp sequence, including six exons and five introns, containing a 1,407 bp ORF encoding a polypeptide of 469 amino acids (GenBank accession number: MH321826; **Supplementary Figure S2**) with a predicted molecular weight of 52.644 kDa and theoretical isoelectric point of 5.48. Furthermore, similar to other teleost PPARαb proteins, ToPPARαb deduced proteins possess four domains containing an N-terminal hypervariable region (A/B), conserved DNA-binding domain (DBD) (C), flexible hinge domain (D) and ligand-binding domain (LBD) (E/F) (**Figure 1B**). The twelve α-helices (H) and four parts of the β-sheet (S) were predicted by comparison with other vertebrate PPARα proteins, and two zinc finger domains (Amino acid residues located in the C^103^-C^123^ and C^140^-C^157^) were in the DBD.

### Functional Characterization of the ToElovl5 Elongase

**FIGURE 2:**
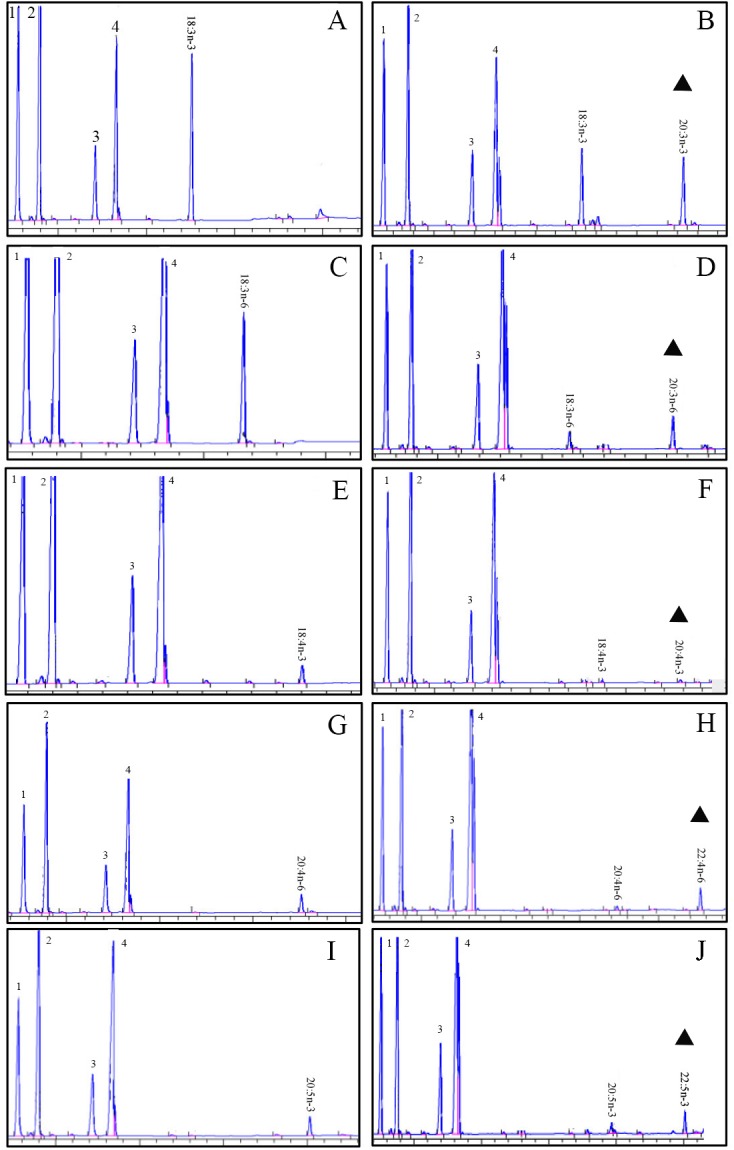
Functional characterization of the putative *Elovl5* in transgenic yeast. Fatty acid methyl esters (FAMEs) were extracted from yeast transformed with the pYES2-Elovl5 and grown in the presence of PUFA substrates 18:3n-3 **(A)**, 18:3n-6 **(C)**, 18:4n-3 **(E)**, 20:4n-6 **(G)**, and 20:5n-3 **(I)**. Based in retention times, additional peaks (marked with a triangular sign) were identified as 20:3n-3 **(B)**, 20:3n-6 **(D)**, 20:4n-3 **(F)**, 22:4n-6 **(H)**, and 22:5n-3 **(J)**. Peaks 1–4 represent the main endogenous FAs of *T. ovatus*, namely C16:0, C16:1 isomers, C18:0 and C18:1n-9, respectively. Raw data are presented in **Supplementary Data Sheets 1–12**.

The role of the *ToElovl5 elongase* in LC-PUFA biosynthesis was investigated by growing transgenic yeast expressing the *ToElovl5* cDNA in the presence of potential PUFA substrates. The results of heterologous expression showed that *ToElovl5* possessed high conversion activity toward C20 PUFA, especially 20:5n-3 (86.6 %) and 20:4n-6 (84.8 %), followed by C18 substrates containing 18:3n-6 (67.4 %), 18:4n-3 (58.3 %), and 18:3n-3 (49.7 %) (**Figure 2** and **Table 1**).

**Table 1:** Conversion rates of pYES2-Elovl5 transformed yeast grown in presence of 18:3n-3, 18:3n-6, 18:4n-3, 20:4n-6, and 20:5n-3 substrates.

FA substrate	Product	Conversion (%)	Activity
18:3n-3	20:3n-3	49.7%	C18→C20
18:3n-6	20:3n-6	67.4%	C18→C20
18:4n-3	20:4n-3	58.3%	C18→C20
20:4n-6	22:4n-6	84.8%	C20→C22
20:5n-3	22:5n-3	86.6%	C20→C22

*Conversions are expressed as a percentage of total FA substrate converted to elongated products*.

### ToPPARαb Structural Analyses

**FIGURE 3:**
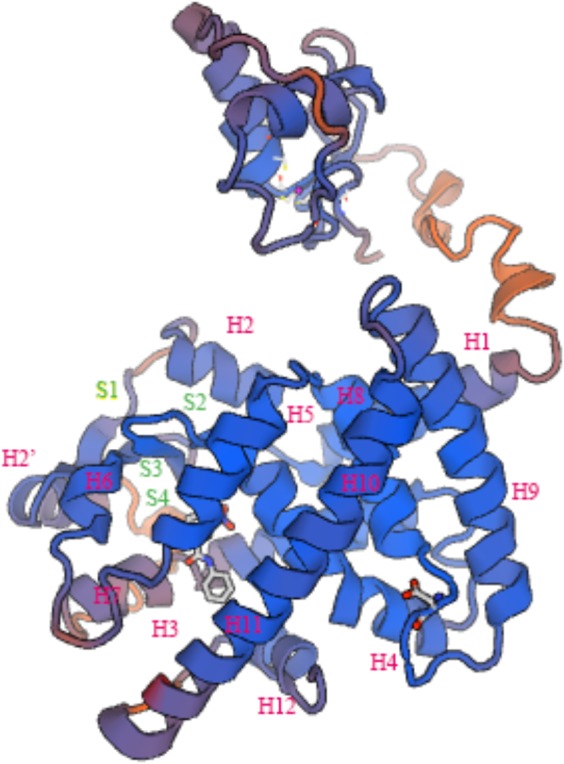
Three-dimensional structures of ToPPARαb deduced amino acid sequences “H” and “S” indicate α-helix and β-sheet, respectively.

**FIGURE 4:**
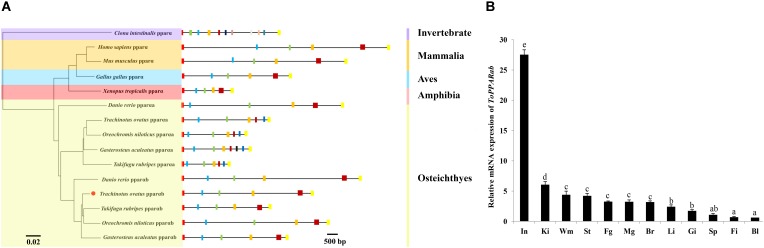
The structure and tissue expression of the *ToPPARαb* gene. **(A)** Genome structure analysis of *PPARα* genes according to the phylogenetic relationship. Lengths of exons and introns of each *PPARα* gene are displayed proportionally. Different color boxes and lines represent exons and introns, respectively. The identical color boxes represent homologous sequences. **(B)** Gene transcription of *ToPPARαb* in various tissues. The twelve tissues are small intestine (In), head-kidney (Ki), white muscle (Wm), stomach (St), female gonad (Fg), male gonad (Mg), brain (Br), liver (Li), gill (Gi), spleen (Sp), fin (Fi), and blood (Bl). The data from different tissues were analyzed by the Duncan test using one-way ANOVA. Data are shown as the means ± SD. Different letters indicate significant differences (*p* < 0.05).

In general, the 3D structure of ToPPARαb was highly similar to that of the *Danio rerio* and *Homo sapiens* homologs (**Figure 3**) (Liang et al., 2016; Ning et al., 2016). Moreover, the genomic structural features of *PPARαb* were further examined in metazoans. The phylogenetic relationship of PPARα in *T. ovatus* and other representative species was constructed (**Figure 4A**). The distribution and lengths of the exons and introns of each *PPARα* gene are also shown in **Supplementary Table S2**. All *PPARαa* and *PPARαb* sequences had seven exons and six introns in fish, except for *Gasterosteus aculeatus* *PPARαa*, which possessed eight exons and seven introns, while *D. rerio PPARαa* possessed six exons. Furthermore, the sizes of homologous intron sequences are different, while the exonic sequences showed nearly no diversity. Moreover, ToPPARαb was grouped together with *Oreochromis niloticus*, which was also in the order Perciformes. The homology with ToPPARα, from close to distant, was other Osteichthyes, Amphibia, Aves, Mammalia, and Invertebrates. This result corresponded with the findings of conventional taxonomy.

### Tissue Expression of ToPPARαb

The tissue expression pattern of *ToPPARαb* was analyzed by qRT-PCR. The *PPARαb* gene was extensively expressed in twelve tissues (**Figure 4B**). The transcription of *ToPPARαb* was tissue specific, and this gene was highly expressed in small intestine and head-kidney, followed by white muscle, stomach, gonads and brain (*P* < 0.05), with lower expression in the spleen, fin and blood (*P* < 0.05).

### PPARαb Positively Promotes ToElovl5 Expression

**FIGURE 5:**
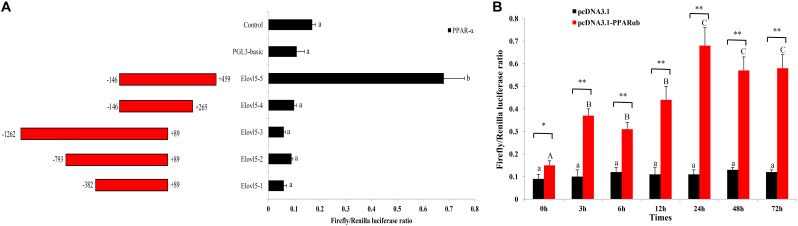
Promoter activity analysis of the *ToElovl5* gene. **(A)** The structure and transcriptional activity of *ToElovl5* promoters. Five recombinant plasmids, denoted Elovl5-1 (–382 to +89), Elovl5-2 (–793 to +89), Elovl5-3 (–1262 to +89), Elovl5-4 (–146 to +265) and Elovl5-5 (–146 to +459) were constructed and transfected with transcription factor PPARαb into HEK 293T cells. **(B)** Dual-luciferase activity driven by the *ToElovl5-5* core promoter upon the transfection of pcDNA3.1-PPAR-α and pcDNA3.1 in HEK 293T cells. All values are presented as the means ± SD (*n* = 3). Asterisks indicate that the values are significantly different from the individual controls (^∗^*p* < 0.05 and ^∗∗^*p* < 0.01). Bars on the same group with different letters are statistically significant from one another (*p* < 0.05).

A total of 1,721 bp of the 5′ flanking sequence of the *Elovl5* gene was cloned and defined as the candidate promoter. To determine the promoter activity of *ToElovl5* with the transcription factor PPARαb in HEK 293T cells, a series of progressive deletion constructs were made (**Figure 5A**). Compared with the activity of the promoter candidate (Elovl5-4), a deletion of fragment from -146 bp to +459 bp (Elovl5-5) increased promoter activity with PPARαb. The expression levels of Elovl5-5 were 6.8-fold greater than those of Elovl5-4 with PPARαb (**Figure 5A**), suggesting that the core promoter region was located at +265 bp to +459 bp, which contained the PPARαb binding sites. To further confirm the interaction of ToPPARαb with *ToElovl5*, the influence of ToPPARαb overexpression on *ToElovl5* transcription was determined. PPARαb overexpression increased the promoter activity of ToElovl5-5 at all tested time points in heterologous HEK 293T cells, and the maximum difference occurred at 24 h posttransfection, which was detected as 6.2-fold higher in PPARαb-overexpressing cells than that in the controls (**Figure 5B**). These results indicated that constitutively expressed PPARαb positively regulated *ToElovl5* expression in HEK 293T cells.

### ToPPARαb Knockdown Decreased ToElovl5 Transcription in TOCF Cells

**FIGURE 6:**
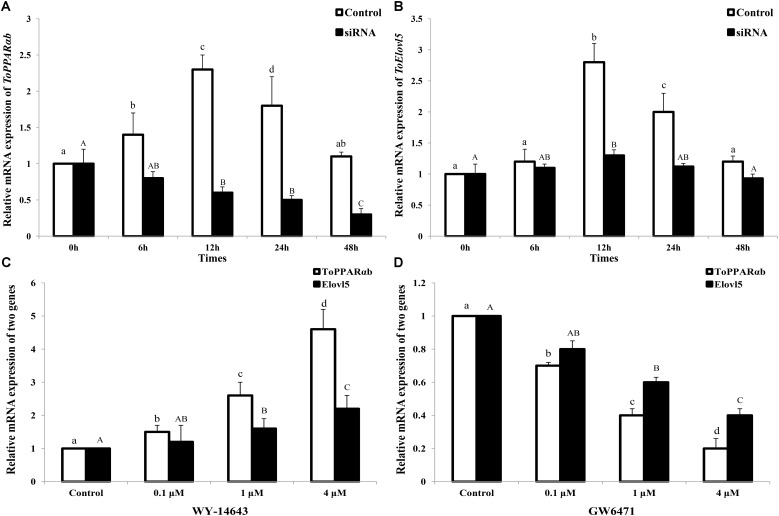
*ToPPARαb* **(A)** and *ToElovl5* **(B)** mRNA expression levels by qRT-PCR after the transfection of either control RNA (control) or siRNA (RNAi). TOCF cells were stimulated with 0.1, 1, and 4 mM of PPARαb agonist (WY-14643) **(C)** and inhibitor (GW6471) **(D)** for 24 h, and the expression levels of *ToPPARαb* and *ToElovl5* were significantly increased and decreased, respectively, in a concentration-dependent manner. All values are expressed as the means ± SD (*n* = 3). Bars on the same group with different letters are statistically significant from one another (*p* < 0.05).

In addition to the above results in HEK 293T cells, the function of PPARαb on *Elovl5* was further confirmed in TOCF cells (**Figures 6A,B**). In the RNAi experiment, the mRNA expression of *ToPPARαb* was drastically reduced in a time-dependent manner, except at 0 h, suggesting the effective knockdown of *ToPPARαb* expression. When *ToPPARαb* mRNA was depleted, *ToElovl5* transcription was significantly repressed compared with the control at the corresponding time points. This result demonstrated a positive regulatory role for *ToPPARαb* on *ToElovl5* mRNA expression in the native *T. ovatus* host.

### The Expression of Elovl5 Was Monitored by the Specific Inhibition and Activation of PPARαb

After stimulation for 24 h, the mRNA expression of ToPPARαb was drastically increased by a PPARαb activator (WY-14643) and memorably decreased by an inhibition (GW6471) in a concentration-dependent manner (**Figures 6C,D**). Moreover, both *ToPPARαb* and *ToElovl5* showed the same expression trend. The mRNA levels of *Elovl5* (*P* < 0.05) dramatically increased with the addition of the PPARαb activator (**Figure 6C**), nevertheless the expression of *Elovl5* was suppressed after addition of the PPARαb inhibitor (**Figure 6D**) in a concentration-dependent manner. These results demonstrated that ToPPARαb played a positive regulatory role in *ToElovl5* transcription in *T. ovatus*.

## Discussion

*Trachinotus ovatus* is widely cultured because of its great commercial value in China. Recently, a study investigating the LC-PUFA content in *T. ovatus* muscle showed that high retention of LC-PUFA occurred in muscle (Zhang et al., 2010). Elongases play core roles in the biosynthesis of LC-PUFA in fish (Castro et al., 2016). Consequently, a better understanding of the potential regulating mechanisms for the transcription of *Elovl5* elongase would conduce to improve the endogenous LC-PUFA synthetic ability of the *T. ovatus*.

Similar to other teleost Elovl5 proteins, the isolated *T. ovatus* Elovl5 possessed all the features of the elongase family including a histidine box (HXXHH), canonical C-terminal ER retrieval signal (KXRXX), and transmembrane domains, supporting its role in LC-PUFA biosynthesis (Jakobsson et al., 2006; Monroig et al., 2012; Xie et al., 2016). The *ToElovl5* could efficiently elongate C18 (18:3n-3, 18:3n-6, and 18:4n-3) and C20 (20:4n-6 and 20:5n-3) substrates to C20 and C22 PUFA, respectively, consistent with previously reported specificities in mammal (Leonard et al., 2000) and teleost (Hastings et al., 2005; Zheng et al., 2009; Gregory et al., 2010; Mohd-Yusof et al., 2010; Morais et al., 2011; Castro et al., 2016), clearly demonstrating that vertebrate *Elovls* universally had extensive substrate specificity. Furthermore, the *Siganus canaliculatus* *Elovl5* had a predilection for n-3 over n-6 PUFA substrates, which was similar to that in most species studied previously, containing both freshwater and marine fish (Mohd-Yusof et al., 2010; Morais et al., 2011).

Additionally, previous studies found that LC-PUFA and their metabolites can regulate transcription of lipid metabolism related genes through modulation of transcription factors including, among others, PPARs (Sampath and Ntambi, 2005). Thus far, three major types of PPARs have been identified, namely, PPARα/β/γ. PPARα is the major PPAR subtype found in hepatocytes and is involved in the regulation of lipid and carbohydrate metabolism genes. Three PPARs function by dimerization with the retinoid X receptor (RXR) and binding to a prescribed DNA sequence, termed the PPAR response element (PPRE) (Desvergne and Wahli, 1999). Similar to PPARα in other species, the ToPPARαb amino acid sequence revealed four representative domains. The DBD domain, the most conserved domain in PPARs, comprises two zinc finger-like motifs folded in a circular structure that identifies the DNA target sequence AGGNCA, and the binding of the PPAR/RXR heterodimer to the PPRE regulates the target gene (Ijpenberg et al., 1997). Analysis of the *ToElovl5* promoter region revealed the presence of typical binding sites of PPARα and Elovl5, and putative binding sites of between ToPPARαb and the *ToElovl5* promoter region need further verification. Nevertheless, the regulatory mechanism of *ToElovl5* is complex. PPARαb is one of the important factors for the increased expression of *ToElovl5* in *T. ovatus*.

Based on the tissue expression profile of *ToPPARαb*, high mRNA levels were detected in metabolically active adipose tissues containing fatty acids, such as intestine, kidney, muscle, stomach, gonads and brain. A similar expression pattern was determined in several other marine fish species, such as *Liza haematocheila, O. niloticus*, and *Lateolabrax japonicus*, which also showed limited LC-PUFA biosynthesis capacity (Dong et al., 2015; Ning et al., 2016; Yang et al., 2017). Since these tissues are major metabolic sites for LC-PUFA (Agbaga et al., 2010), it was reasonable that the *ToPPARαb* gene showed relatively high expression.

Numerous studies have shown that PPARα was necessary for the clofibrate stimulation of peroxisomal and microsomal enzymes, such as acyl-CoA oxidase (AOX) (Berthou et al., 1995), the rate-limiting enzyme for fatty acid β-oxidation (Brandt et al., 1998), SREBP-1c (Yoshikawa et al., 2003) and fatty acid transport proteins and translocases in the liver (Frohnert et al., 1999). Moreover, PPARs are ligand-activated transcription factors that regulate gene expression in the PUFAs biosynthesis pathway (Sampath and Ntambi, 2005). In the present study, the positive regulatory role of ToPPARαb in *ToElovl5* transcription in *T. ovatus* was characterized. The results of the luciferase reporter assay, as well as RNAi analysis, clearly demonstrated that *ToElovl5* expression was regulated by PPARαb in *T. ovatus* (**Figures 5**, **6A,B**). These results provided the first evidence of the involvement of PPARαb in the expression of the rate-limiting enzyme *Elovl5*. *ToElovl5* transcription indicated increasing profiles in either native TOCF cells or heterologous HEK 293T cells. These results were reasonable due to the stress caused by the disturbed biological environment during *in vitro* TOCF cell culture or *Elovl5* promoter expression in the heterologous host (Liu et al., 2018).

To further determine the transcription mechanism of ToPPARαb in *T. ovatus*, the mRNA levels of *ToPPARαb* and *ToElovl5* were detected. The transcription of *ToPPARαb* and *ToElovl5* was prominently increased or decreased in a concentration-dependent manner of activator or inhibition, respectively (**Figures 6C,D**). This observation was consistent with the results of studies implemented in mammals (Wang et al., 2005, 2006), suggesting that *ToPPARαb* could up-regulate *ToElovl5* in fish. The results of the *in vitro* experiment in the present study confirmed the above findings by over-expression and suppression of *ToPPARαb*. These results verified the direct stimulatory role of PPARαb on *Elovl5* and suggested that such regulatory mechanisms operated differently compared to mammals.

In general, structural complexity was caused by intron gain or loss, which is a core evolutionary mechanism in most gene families (Yu et al., 2018). An exon–intron structure analysis of the *ToPPARαb* gene indicated that all *PPARαb* genes had six exons, while *PPARαa* had seven exons in fish, except *G. aculeatus* *PPARαa*, which possessed eight exons, and *D. rerio PPARαa*, which possessed six exons. These findings might represent introns gained or lost during evolution and may also suggest that the metazoan *PPARα* genes consisted of highly conserved numbers of exons and introns. The results of the phylogenetic analysis were consistent with the findings of conventional taxonomy, suggesting that *ToPPARα* exhibited a closer genetic relationship with Perciformes, such as *O. niloticus* *PPARα*.

In summary, we demonstrated clear associations between PPARαb and the *ToElovl5* promoter, as well as the positive regulatory functions of PPARαb in *ToElovl5* transcription in *T. ovatus*. Moreover, the proposed synthesis pathway of LC-PUFA in *T. ovatus* (**Supplementary Figure S3**). The present study provided the first evidence of a positive regulator of *ToElovl5* transcription. It would be interesting to further clarify the interactions between PPARαb and the proposed cooperative companions to better comprehend the mechanisms underlying the PPARαb-mediated regulation of *ToElovl5* transcription. Furthermore, the specific mechanism of PPARαb in regulating *ToElovl5* by directly binding or being assisted by other proteins still needs further investigation.

## Author Contributions

K-CZ, S-GJ, and D-CZ designed the research and wrote the paper. LS, C-PZ, and K-CZ performed the research. H-YG and NZ analyzed the data. B-SL and LG contributed reagents, materials, and analysis tools.

## Conflict of Interest Statement

The authors declare that the research was conducted in the absence of any commercial or financial relationships that could be construed as a potential conflict of interest. The handling Editor declared a shared affiliation, though no other collaboration, with several of the authors C-PZ, LS at time of review.

## Footnotes

1 http://blast.ncbi.nlm.nih.gov/Blast.cgi

2 http://asia.ensembl.org/

3 http://genome.ucsc.edu/cgi-bin/hgBlat

4 http://www.cbs.dtu.dk/services/SignalP/

5 http://web.expasy.org/protparam/

6 http://www.ebi.ac.uk/Tools/msa/clustalw2/

## Supplementary Material

FIGURE S1 The nucleotide sequence of *Elovl5* gene and the deduced amino acid sequence of *Trachinotus ovatus*. Initiation and termination codons are marked by box. The structure and functional domains is underlined. Yellow marked endoplasmic reticulum retention signal.

FIGURE S2 The nucleotide sequence of *PPARαb* gene and the deduced amino acid sequence of *Trachinotus ovatus*. Initiation and termination codons are marked by red. Yellow boxes indicate the two zinc finger domains (amino acid residues located in the C^103^-C^123^ and C^140^-C^157^) were in DBD.

FIGURE S3 The proposed synthesis pathway of PUFA in *T. ovatus*. Red arrows represent the pathway confirmed in *T. ovatus*.

TABLE S1 Primers used for sequence cloning, deletion mutant construction, mRNA construction and qRT-PCR.

TABLE S2 Lengths of exons and introns of each *PPARα* gene.

TABLE S3 PPARα and Elovl5 proteins used in multiple alignment.
