# Supplementary figures and images for: The Transcriptional Factor PPARαb Positively Regulates Elovl5 Elongase in Golden Pompano Trachinotus ovatus (Linnaeus 1758)
Source: Front Physiol. 2018 Sep 25;9:1340. doi: 10.3389/fphys.2018.01340 (PMC6167968; doi:10.3389/fphys.2018.01340)

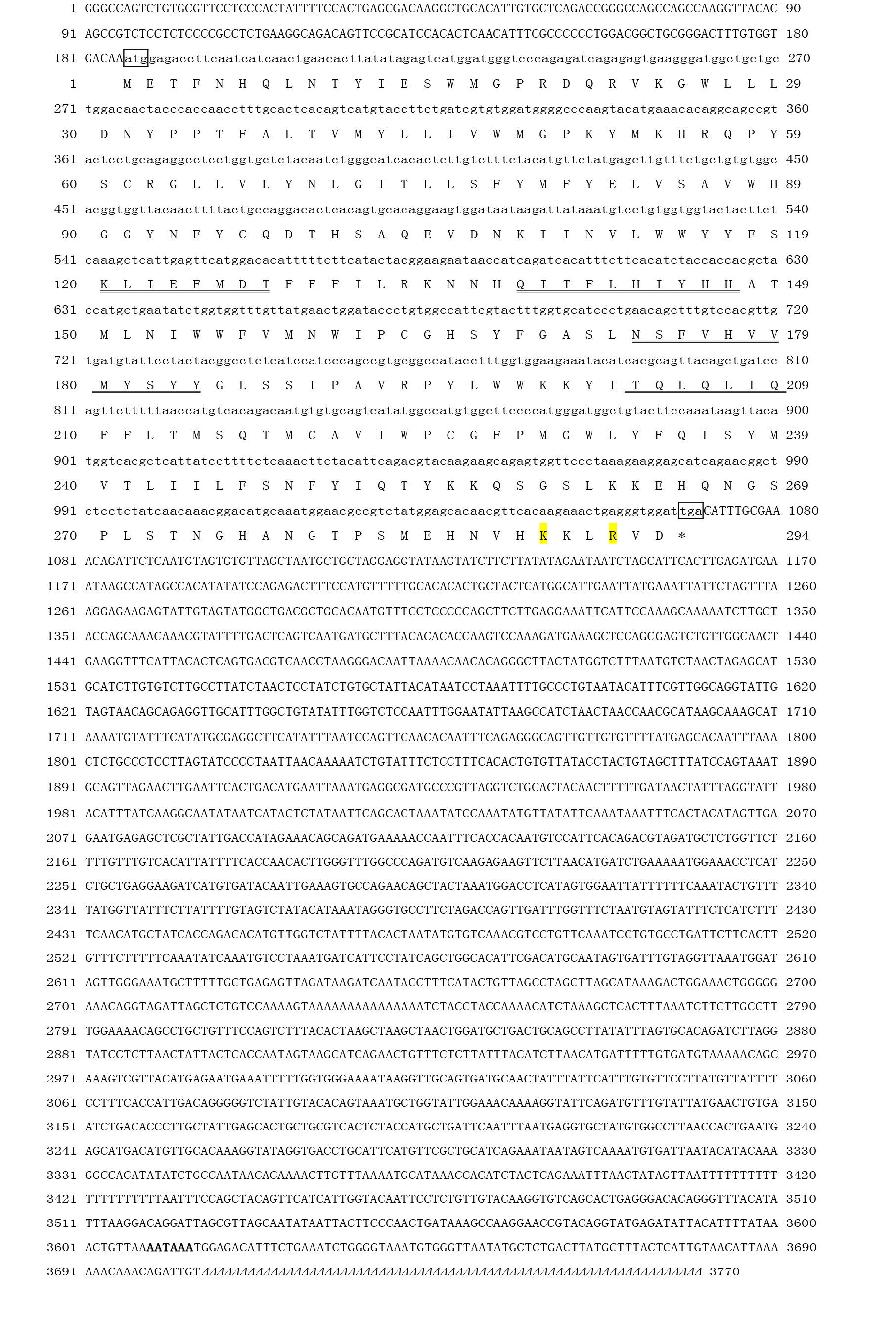

Supplement: FIGURE S1 — The nucleotide sequence of Elovl5 gene and the deduced amino acid sequence of Trachinotus ovatus. Initiation and termination codons are marked by box. The structure and functional domains is underlined. Yellow marked endoplasmic reticulum retention signal. [file Image_1.jpg]

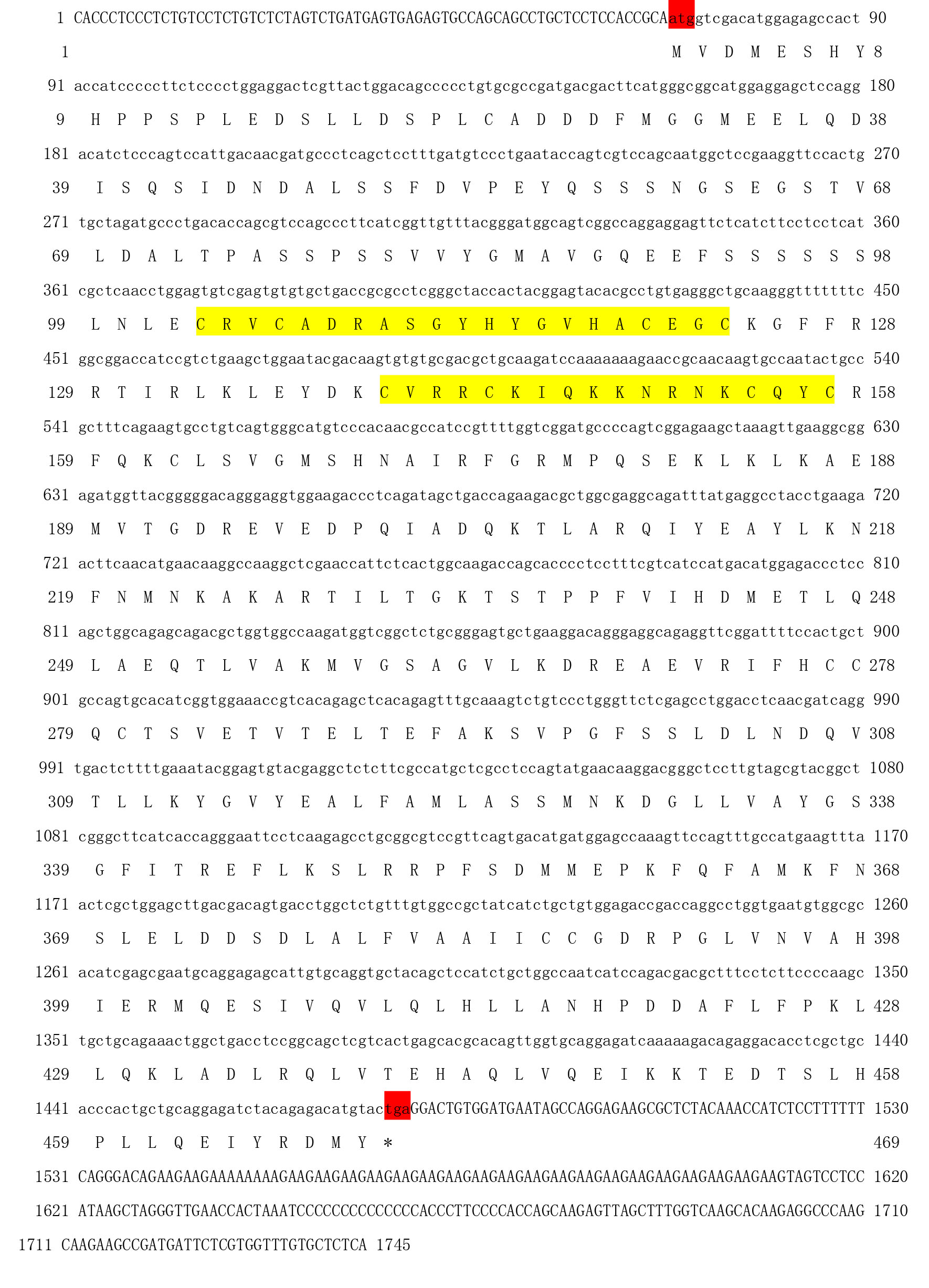

Supplement: FIGURE S2 — The nucleotide sequence of PPARαb gene and the deduced amino acid sequence of Trachinotus ovatus. Initiation and termination codons are marked by red. Yellow boxes indicate the two zinc finger domains (amino acid residues located in the C103-C123 and C140-C157) were in DBD. [file Image_2.jpg]

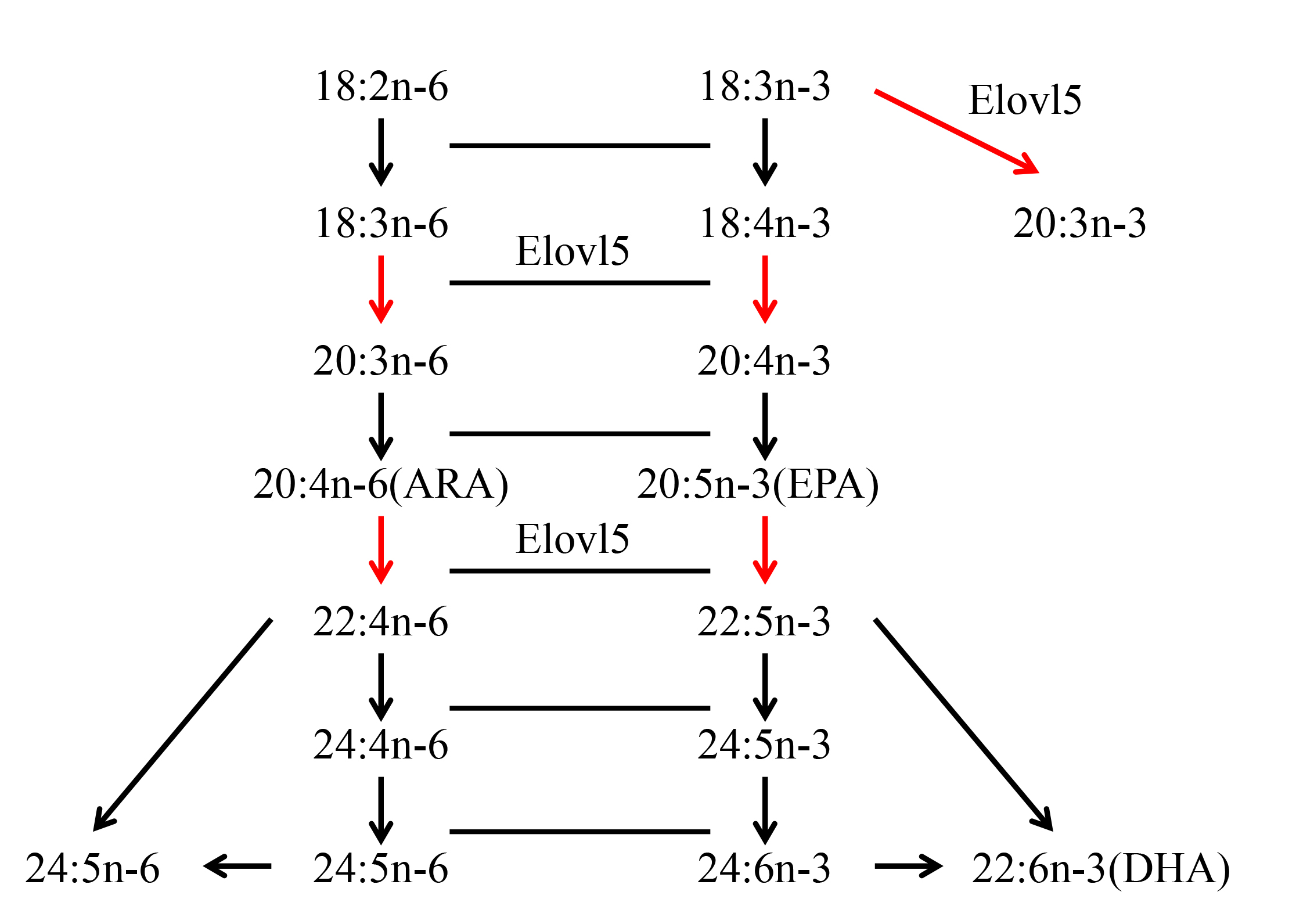

Supplement: FIGURE S3 — The proposed synthesis pathway of PUFA in T. ovatus. Red arrows represent the pathway confirmed in T. ovatus. [file Image_3.jpg]

# PPARab

0h

6h

12h

24h

PPARab-control

PPARab-siRNA

GAPDH

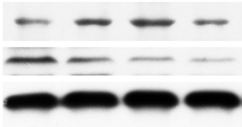

Supplement: Supplementary file 15 [file Data_Sheet_11.PDF]
