# Supplementary material for: The Transcriptional Factor PPARαb Positively Regulates Elovl5 Elongase in Golden Pompano Trachinotus ovatus (Linnaeus 1758)
Source: Front Physiol. 2018 Sep 25;9:1340. doi: 10.3389/fphys.2018.01340 (PMC6167968; doi:10.3389/fphys.2018.01340)
Supplement: TABLE S1 — Primers used for sequence cloning, deletion mutant construction, mRNA construction and qRT-PCR. [file Table_1.docx]

**Supplementary Table 1**. Primers used for sequence cloning, deletion mutant construction, mRNA construction and qRT-PCR.

| **Subject and Primers** | **Nucleotide sequence** |
| --- | --- |
| **Primers for sequence cloning** |  |
| PPARαb-ORF-F | CTAGCTAGCATGGTCGACATGGAGAGCCAC |
| PPARαb-ORF-R | CCCAAGCTTTCAGTACATGTCTCTGTA |
| Elovl5-ORF-F | CCCAAGCTTATGGAGACCTTCAATCATCAACTG |
| Elovl5-ORF-R | CCGCTCGAGTCAATCCACCCTCAGTTTCTTG |
| Elovl5-3’outer1 | GGCGATGCCCGTTAGGTCTG |
| Elovl5-3’outer2 | GCACTGCTGCGTCACTCTACC |
| **Deletion mutant construction** |  |
| Elovl5-pF1 | CGGGGTACCTGTACAAACAATTTAAG |
| Elovl5-pF1 | CCGCTCGAGAAATGTGGCCACTTCTCA |
| Elovl5-pF2 | CGGGGTACCGGACTGTGTAAATACCTC |
| Elovl5-pF2 | CCGCTCGAGAAATGTGGCCACTTCTCA |
| Elovl5-pF3 | CGGGGTACCTTCTGTTAACTCGTAAAGC |
| Elovl5-pF3 | CCGCTCGAGAAATGTGGCCACTTCTCA |
| Elovl5-pF4 | CGGGGTACCCACAAAGTAAACGTTGT |
| Elovl5-pF4 | CCGCTCGAGTGACTAAAACCTGTCAA |
| Elovl5-pF5 | CGGGGTACCCACAAAGTAAACGTTGT |
| Elovl5-pF5 | CCGCTCGAGATACTGTTTTCAGGTGACAA |
| **Primers for qRT-PCR** |  |
| qRT-E5-F | ATGGTCACGCTCATTATCCT |
| qRT-E5-R | ATGTCAATCCACCCTCAGTT |
| qRT-PPARα-F | AGTGACCTGGCTCTGTTTG |
| qRT-PPARα-R | AAGCGTCGTCTGGATGATT |
| EF1α-F | AAGCCAGGTATGGTTGTCAACTTT |
| EF1α-R | CGTGGTGCATCTCCACAGACT |
| **siRNA** |  |
| PPARαb-si | GCGAATGCAGGAGAGCATT |
| PPARαb-NC | ACGUGACACGUUCGGAGAATT |

**Supplementary Table 2** Lengths of exons and introns of each *PPARα* gene.

|  | E1 | I1 | E2 | I2 | E3 | I3 | E4 | I4 | E5 | I5 | E6 | I6 | E7 | I7 | E8 | I8 | E9 | I9 | E10 | I10 | E11 |
| --- | --- | --- | --- | --- | --- | --- | --- | --- | --- | --- | --- | --- | --- | --- | --- | --- | --- | --- | --- | --- | --- |
| *Ciona intestinalis* pparα | 117 | 395 | 93 | 355 | 138 | 73 | 201 | 204 | 105 | 545 | 669 | 434 | 126 | 1821 | 126 | 482 | 117 | 266 | 102 | 1458 | 138 |
| *Homo sapiens* pparα | 207 | 16581 | 162 | 2929 | 138 | 1410 | 204 | 11777 | 447 | 2893 | 243 |  |  |  |  |  |  |  |  |  |  |
| *Mus musculus* pparα | 207 | 9340 | 162 | 1710 | 138 | 1712 | 204 | 6773 | 447 | 2747 | 246 |  |  |  |  |  |  |  |  |  |  |
| *Gallus gallus* pparα | 207 | 2525 | 162 | 1247 | 138 | 2411 | 204 | 2469 | 447 | 1514 | 246 |  |  |  |  |  |  |  |  |  |  |
| *Xenopus tropicalis* pparα | 228 | 1075 | 162 | 350 | 138 | 644 | 201 | 538 | 447 | 253 | 246 |  |  |  |  |  |  |  |  |  |  |
| *Danio rerio* pparαa | 201 | 503 | 159 | 7828 | 138 | 6664 | 201 | 2254 | 468 | 3926 | 243 |  |  |  |  |  |  |  |  |  |  |
| *Trachinotus ovatus* pparαa | 183 | 986 | 156 | 2853 | 138 | 1572 | 201 | 112 | 261 | 114 | 243 | 98 | 243 |  |  |  |  |  |  |  |  |
| *Oreochromis niloticus* pparαa | 183 | 373 | 156 | 2638 | 138 | 782 | 201 | 86 | 261 | 88 | 243 | 103 | 243 |  |  |  |  |  |  |  |  |
| *Gasterosteus aculeatus* pparαa | 183 | 928 | 156 | 2113 | 138 | 725 | 201 | 96 | 67 | 61 | 150 | 146 | 243 | 108 | 243 |  |  |  |  |  |  |
| *Takifugu rubripes* pparαa | 234 | 78 | 159 | 1416 | 138 | 438 | 201 | 73 | 264 | 74 | 243 | 385 | 243 |  |  |  |  |  |  |  |  |
| *Danio rerio* pparαb | 189 | 3258 | 159 | 4697 | 138 | 5956 | 189 | 9115 | 459 | 581 | 243 |  |  |  |  |  |  |  |  |  |  |
| *Trachinotus ovatus* pparαb | 207 | 2402 | 168 | 1751 | 138 | 4139 | 201 | 3318 | 450 | 234 | 243 |  |  |  |  |  |  |  |  |  |  |
| *Takifugu rubripes* pparαb | 207 | 1153 | 162 | 1269 | 141 | 1871 | 201 | 1252 | 453 | 81 | 243 |  |  |  |  |  |  |  |  |  |  |
| *Oreochromis niloticus* pparαb | 204 | 2201 | 168 | 4494 | 138 | 4143 | 201 | 2584 | 453 | 96 | 243 |  |  |  |  |  |  |  |  |  |  |
| *Gasterosteus aculeatus* pparαb | 207 | 1465 | 165 | 1553 | 138 | 2296 | 201 | 2784 | 453 | 199 | 243 |  |  |  |  |  |  |  |  |  |  |

**Supplementary Table 3**

PPARα and Elovl5 proteins used in multiple alignment.

| **Species** | **Protein name** | **GenBank No.** | **Species** | **Protein name** | **GenBank No.** |
| --- | --- | --- | --- | --- | --- |
| *Trachinotus ovatus* | PPARαb | MH321826 | *Oreochromis niloticus* | Elovl5 | NP_001266389.1 |
| *Danio rerio* | PPARαa | NP_001154805.1 | *Trachinotus ovatus* | Elovl5 | KY860144 |
| *Danio rerio* | PPARαb | NP_001096037.1 | *Gallus gallus* | Elovl5 | XP_015140326.1 |
| *Scophthalmus maximus* | PPARαb | AGC31488.1 | *Homo sapiens* | Elovl5 | NP_001229759.1 |
| *Lateolabrax japonicus* | PPARαb | ACI42381.1 | *Oryzias latipes* | Elovl5 | XP_004077464.1 |
| *Homo sapiens* | PPARα | NP_005027.2 | *Gasterosteus aculeatus* | Elovl5 | ENSGACP00000008519 |
| *Mus musculus* | PPARα | EDL04428.1 | *Tetraodon nigroviridis* | Elovl5 | CAG09412.1 |
| *Oreochromis niloticus* | PPARαb | XP_003443968.1 | *Danio rerio* | Elovl5 | NP_956747.1 |
| *Gadus morhua* | Elovl5 | AAT81406.1 | *Petromyzon marinus* | Elovl5 | ALZ50286.1 |
| *Ornithorhynchus anatinus* | Elovl5 | XP_007658623.1 | *Xenopus tropicalis* | Elovl5 | NP_001011248.1 |
