# Supplementary material for: The Transcriptional Factor PPARαb Positively Regulates Elovl5 Elongase in Golden Pompano Trachinotus ovatus (Linnaeus 1758)
Source: Front Physiol. 2018 Sep 25;9:1340. doi: 10.3389/fphys.2018.01340 (PMC6167968; doi:10.3389/fphys.2018.01340)
Supplement: Supplementary file 5 [file Data_Sheet_1.PDF]

数据文件: C:\CHEM32\1\DATA\201701\DEF_GC 2017-01-16 09-51-36\122F0801.D
样品名称: BW4482-18-1

===

操作者	: asp	序列行 :	8
仪器	: 仪器 1	位置 :	样品瓶 122
进样日期	: 2017/1/16 15:41:07	进样次数 :	1
		进样量 :	1 µl

采集方法 : C:\CHEM32\1\DATA\201701\DEF_GC 2017-01-16 09-51-36\FID-脂肪酸HP88-NEW.M
最后修改 : 2017/1/12 14:35:37 : asp
分析方法 : C:\CHEM32\1\METHODS\FID-肉桂酸.M
最后修改 : 2017/3/28 10:30:28 : asp
(调用后修改)
附加信息: 峰已手动积分

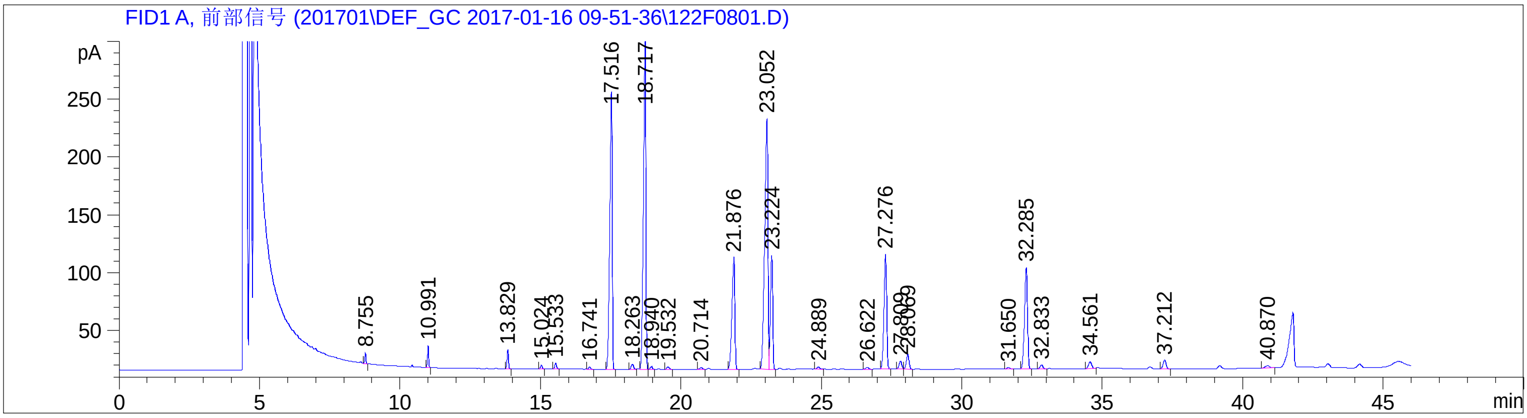

===
面积百分比报告
===

排序 : 信号
乘积因子: : 1.0000
稀释因子: : 1.0000
内标使用乘积因子和稀释因子

信号 1: FID1 A, 前部信号

峰 #	保留时间 [min]	类型	峰宽 [min]	峰面积 [pA*s]	峰高 [pA]	峰面积 %
1	8.755	BB	0.0444	24.59929	9.43199	0.30835
2	10.991	BB	0.0465	55.51491	18.74943	0.69587
3	13.829	BB	0.0621	61.66692	16.29840	0.77299
4	15.024	BB	0.0663	13.21402	3.19409	0.16564
5	15.533	BB	0.0720	22.29717	5.00294	0.27949
6	16.741	BB	0.0777	8.30358	1.73768	0.10408
7	17.516	BB	0.0902	1433.14526	238.32285	17.96430
8	18.263	BB	0.0918	26.36135	4.53754	0.33044
9	18.717	BV	0.0878	1743.31091	291.29977	21.85218
10	18.940	VB	0.0737	11.88381	2.48817	0.14896
11	19.532	BB	0.0888	12.98546	2.33612	0.16277
12	20.714	BB	0.0931	10.46721	1.71634	0.13121
13	21.876	BB	0.1094	687.01855	96.13190	8.61169
14	23.052	BV	0.1224	1712.04102	216.12746	21.46022
15	23.224	VB	0.0869	545.21808	98.07011	6.83424
16	24.889	BB	0.1011	12.93802	2.06489	0.16218
17	26.622	BB	0.1092	11.54327	1.61906	0.14469
18	27.276	BB	0.1037	655.16132	98.32207	8.21237

峰 #	保留时间 [min]	类型	峰宽 [min]	峰面积 [pA*s]	峰高 [pA]	峰面积 %
19	27.809	BV	0.1096	47.96077	7.03170	0.60118
20	28.069	VB	0.1035	85.02757	12.80702	1.06581
21	31.650	BB	0.1036	7.86208	1.21375	0.09855
22	32.285	BB	0.1141	646.30194	87.53330	8.10131
23	32.833	BB	0.1076	23.80233	3.48859	0.29836
24	34.561	BB	0.1251	44.10058	5.64694	0.55280
25	37.212	BB	0.1194	55.31014	7.38035	0.69331
26	40.870	BB	0.1618	19.70636	1.91921	0.24702

总量 : 7977.74194 1234.47167

==

*** 报告结束 ***
