# Supplementary material for: The Transcriptional Factor PPARαb Positively Regulates Elovl5 Elongase in Golden Pompano Trachinotus ovatus (Linnaeus 1758)
Source: Front Physiol. 2018 Sep 25;9:1340. doi: 10.3389/fphys.2018.01340 (PMC6167968; doi:10.3389/fphys.2018.01340)
Supplement: Supplementary file 6 [file Data_Sheet_2.PDF]

数据文件: C:\CHEM32\1\DATA\201701\DEF_GC 2017-01-16 09-51-36\122F0901.D
样品名称: BW4482-18-2

===

操作者	: asp	序列行 :	9
仪器	: 仪器 1	位置 :	样品瓶 122
进样日期	: 2017/1/16 16:30:45	进样次数 :	1
		进样量 :	1 µl

采集方法 : C:\CHEM32\1\DATA\201701\DEF_GC 2017-01-16 09-51-36\FID-脂肪酸HP88-NEW.M
最后修改 : 2017/1/12 14:35:37 : asp
分析方法 : C:\CHEM32\1\METHODS\FID-肉桂酸.M
最后修改 : 2017/3/28 10:30:28 : asp
(调用后修改)
附加信息: 峰已手动积分

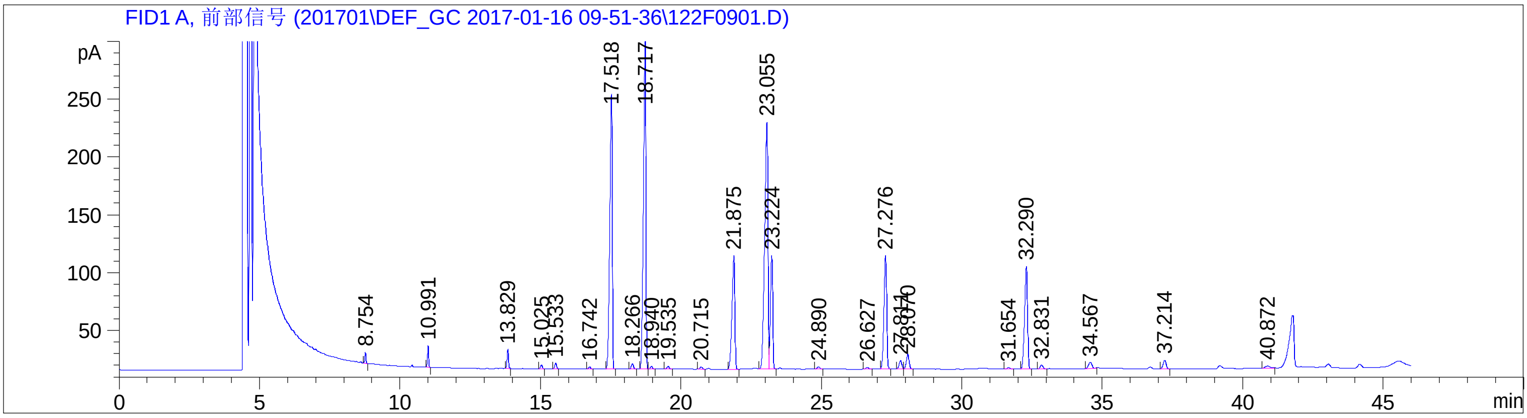

===
面积百分比报告
===

排序 : 信号
乘积因子: : 1.0000
稀释因子: : 1.0000
内标使用乘积因子和稀释因子

信号 1: FID1 A, 前部信号

峰 #	保留时间 [min]	类型	峰宽 [min]	峰面积 [pA*s]	峰高 [pA]	峰面积 %
1	8.754	BB	0.0442	24.79550	9.57066	0.30874
2	10.991	BB	0.0468	55.71205	18.66422	0.69370
3	13.829	BB	0.0599	62.00231	16.49226	0.77202
4	15.025	BB	0.0667	13.56209	3.25170	0.16887
5	15.533	BB	0.0697	22.25452	5.02476	0.27710
6	16.742	BB	0.0741	8.36910	1.80420	0.10421
7	17.518	BB	0.0954	1443.45520	235.96468	17.97321
8	18.266	BB	0.0883	24.63861	4.46954	0.30679
9	18.717	BV	0.0930	1751.12756	287.58414	21.80420
10	18.940	VB	0.0749	11.50780	2.44772	0.14329
11	19.535	BB	0.0931	13.20221	2.29515	0.16439
12	20.715	BB	0.0925	10.46055	1.73006	0.13025
13	21.875	BB	0.1099	690.14862	98.33537	8.59340
14	23.055	FM R	0.1351	1731.49988	213.57460	21.55980
15	23.224	VB	0.0895	550.02411	98.02710	6.84864
16	24.890	BB	0.1024	13.52378	2.06510	0.16839
17	26.627	BB	0.1083	11.52797	1.63354	0.14354
18	27.276	BB	0.1045	658.52612	97.86239	8.19965

数据文件: C:\CHEM32\1\DATA\201701\DEF_GC 2017-01-16 09-51-36\122F0901.D
样品名称: BW4482-18-2

峰 #	保留时间 [min]	类型	峰宽 [min]	峰面积 [pA*s]	峰高 [pA]	峰面积 %
19	27.811	BV	0.1084	48.60561	7.05228	0.60521
20	28.070	VB	0.1064	85.61826	12.73668	1.06608
21	31.654	BB	0.1040	7.46260	1.17624	0.09292
22	32.290	BB	0.1154	648.51416	88.62286	8.07499
23	32.831	BB	0.1096	23.62213	3.46379	0.29413
24	34.567	BB	0.1361	45.87560	5.34751	0.57122
25	37.214	BB	0.1132	55.52602	7.60076	0.69138
26	40.872	BB	0.1653	19.58605	1.82407	0.24388
总量 :				8031.14842	1228.62138	

===

*** 报告结束 ***
