# Supplementary material for: The Transcriptional Factor PPARαb Positively Regulates Elovl5 Elongase in Golden Pompano Trachinotus ovatus (Linnaeus 1758)
Source: Front Physiol. 2018 Sep 25;9:1340. doi: 10.3389/fphys.2018.01340 (PMC6167968; doi:10.3389/fphys.2018.01340)
Supplement: Supplementary file 7 [file Data_Sheet_3.PDF]

数据文件: C:\CHEM32\1\DATA\201701\DEF_GC 2017-01-16 09-51-36\123F1001.D
样品名称: BW4482-19-1

===

操作者 : asp　　　　序列行 : 10
仪器 : 仪器 1　　　　位置 : 样品瓶 123
进样日期 : 2017/1/16 17:20:18　　　　进样次数 : 1
进样量 : 1 µl
采集方法 : C:\CHEM32\1\DATA\201701\DEF_GC 2017-01-16 09-51-36\FID-脂肪酸HP88-NEW.M
最后修改 : 2017/1/12 14:35:37 : asp
分析方法 : C:\CHEM32\1\METHODS\FID-肉桂酸.M
最后修改 : 2017/3/28 10:30:28 : asp
(调用后修改)
附加信息: 峰已手动积分

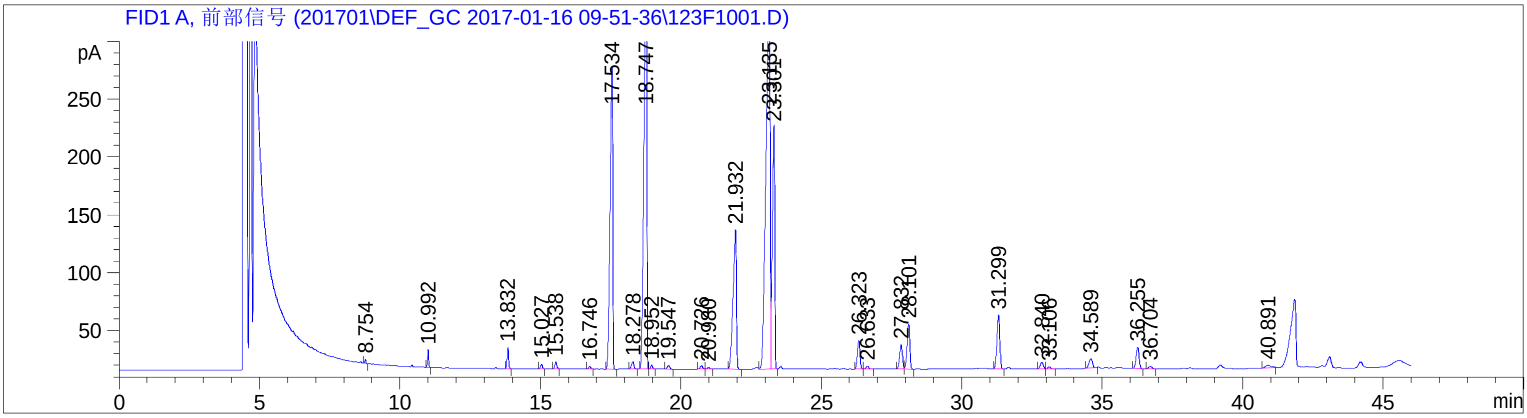

===

面积百分比报告

===

排序 : 信号
乘积因子: : 1.0000
稀释因子: : 1.0000
内标使用乘积因子和稀释因子

信号 1: FID1 A, 前部信号

峰 #	保留时间 [min]	类型	峰宽 [min]	峰面积 [pA*s]	峰高 [pA]	峰面积 %
1	8.754	BB	0.0443	9.28349	3.56883	0.08226
2	10.992	BB	0.0470	45.07484	15.03322	0.39941
3	13.832	BB	0.0620	72.35067	18.36591	0.64109
4	15.027	BB	0.0671	16.22071	3.85541	0.14373
5	15.538	BB	0.0764	27.88368	5.76875	0.24708
6	16.746	BB	0.0761	11.72900	2.43952	0.10393
7	17.534	BB	0.0984	1694.47974	258.72333	15.01467
8	18.278	BB	0.1011	40.53247	6.30014	0.35916
9	18.747	BV	0.0944	2599.18652	396.83566	23.03122
10	18.952	VB	0.0758	15.59652	3.26207	0.13820
11	19.547	BB	0.0953	20.01495	3.27569	0.17735
12	20.726	BV	0.1016	20.18832	3.03607	0.17889
13	20.980	VB	0.0958	9.52442	1.54798	0.08440
14	21.932	BB	0.1221	991.35516	120.37695	8.78433
15	23.135	FM R	0.1708	3265.19702	318.58380	28.93269
16	23.301	VV	0.0942	1221.47913	209.05067	10.82344
17	26.323	BB	0.1011	156.92868	24.37945	1.39053
18	26.633	BB	0.1049	15.88154	2.41059	0.14073

峰 #	保留时间 [min]	类型	峰宽 [min]	峰面积 [pA*s]	峰高 [pA]	峰面积 %
19	27.832	BV	0.1103	147.43452	20.88903	1.30641
20	28.101	VB	0.1064	261.92105	38.99741	2.32086
21	31.299	BB	0.1109	329.99838	46.46202	2.92409
22	32.840	BV	0.1195	43.73680	5.70042	0.38755
23	33.106	VB	0.1159	12.26629	1.70467	0.10869
24	34.589	BB	0.1395	73.53204	8.12886	0.65156
25	36.255	BB	0.1152	137.24423	18.78685	1.21611
26	36.704	BB	0.1266	15.86256	1.91588	0.14056
27	40.891	BB	0.2060	30.59143	2.17638	0.27107

总量 : 1.12855e4 1541.57556

==

*** 报告结束 ***
