# Supplementary material for: The Transcriptional Factor PPARαb Positively Regulates Elovl5 Elongase in Golden Pompano Trachinotus ovatus (Linnaeus 1758)
Source: Front Physiol. 2018 Sep 25;9:1340. doi: 10.3389/fphys.2018.01340 (PMC6167968; doi:10.3389/fphys.2018.01340)
Supplement: Supplementary file 8 [file Data_Sheet_4.PDF]

数据文件: C:\CHEM32\1\DATA\201701\DEF_GC 2017-01-16 09-51-36\123F1101.D
样品名称: BW4482-19-2

===

操作者	: asp	序列行 :	11
仪器	: 仪器 1	位置 :	样品瓶 123
进样日期	: 2017/1/16 18:09:55	进样次数 :	1
		进样量 :	1 µl

采集方法 : C:\CHEM32\1\DATA\201701\DEF_GC 2017-01-16 09-51-36\FID-脂肪酸HP88-NEW.M
最后修改 : 2017/1/12 14:35:37 : asp
分析方法 : C:\CHEM32\1\METHODS\FID-肉桂酸.M
最后修改 : 2017/3/28 10:30:28 : asp
(调用后修改)
附加信息: 峰已手动积分

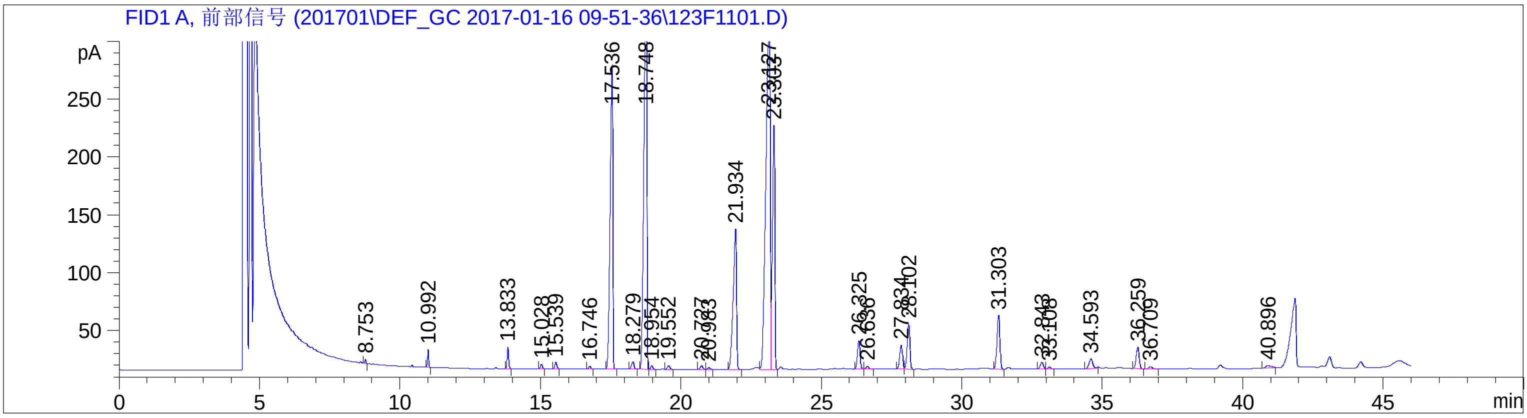

===

面积百分比报告

===

排序 : 信号
乘积因子: : 1.0000
稀释因子: : 1.0000
内标使用乘积因子和稀释因子

信号 1: FID1 A, 前部信号

峰 #	保留时间 [min]	类型	峰宽 [min]	峰面积 [pA*s]	峰高 [pA]	峰面积 %
1	8.753	BB	0.0424	9.53221	3.65808	0.08351
2	10.992	BB	0.0471	45.71886	15.19140	0.40054
3	13.833	BB	0.0619	73.22991	18.61155	0.64156
4	15.028	BB	0.0676	16.70942	3.93192	0.14639
5	15.539	BB	0.0766	28.03079	5.77832	0.24557
6	16.746	BB	0.0763	11.67800	2.41788	0.10231
7	17.536	BB	0.1026	1714.43555	261.24686	15.01994
8	18.279	BB	0.0991	40.37659	6.44239	0.35373
9	18.748	BV	0.0989	2627.91602	388.39081	23.02283
10	18.954	VB	0.0767	15.48297	3.29957	0.13564
11	19.552	BB	0.0979	20.40413	3.21917	0.17876
12	20.727	BB	0.1024	20.58102	3.06194	0.18031
13	20.983	BB	0.0939	9.47725	1.58219	0.08303
14	21.934	BB	0.1206	1001.70728	120.97237	8.77583
15	23.127	FM R	0.1759	3305.82104	313.26877	28.96186
16	23.303	VV	0.0903	1232.58142	210.57939	10.79848
17	26.325	BB	0.1039	158.35231	24.35623	1.38730
18	26.636	BB	0.1050	15.83525	2.39972	0.13873

峰 #	保留时间 [min]	类型	峰宽 [min]	峰面积 [pA*s]	峰高 [pA]	峰面积 %
19	27.834	BV	0.1129	147.84007	20.80731	1.29521
20	28.102	VB	0.1071	264.00916	38.94107	2.31295
21	31.303	BB	0.1123	333.88184	46.22421	2.92509
22	32.843	BV	0.1185	44.47304	5.86142	0.38962
23	33.108	VB	0.1123	12.67852	1.75558	0.11107
24	34.593	BB	0.1485	77.17582	8.15104	0.67613
25	36.259	BB	0.1177	138.54190	18.87075	1.21375
26	36.709	BB	0.1317	16.28571	1.90550	0.14268
27	40.896	BB	0.2075	31.63786	2.17801	0.27718

总量 : 1.14144e4 1533.10343

===

*** 报告结束 ***
