# Supplementary material for: The Transcriptional Factor PPARαb Positively Regulates Elovl5 Elongase in Golden Pompano Trachinotus ovatus (Linnaeus 1758)
Source: Front Physiol. 2018 Sep 25;9:1340. doi: 10.3389/fphys.2018.01340 (PMC6167968; doi:10.3389/fphys.2018.01340)
Supplement: Supplementary file 9 [file Data_Sheet_5.PDF]

数据文件: C:\CHEM32\1\DATA\201701\DEF_GC 2017-01-16 09-51-36\124F1201.D
样品名称: BW4482-20-1

==

操作者	: asp	序列行 :	12
仪器	: 仪器 1	位置 :	样品瓶 124
进样日期	: 2017/1/16 18:59:26	进样次数 :	1
		进样量 :	1 µl

采集方法 : C:\CHEM32\1\DATA\201701\DEF_GC 2017-01-16 09-51-36\FID-脂肪酸HP88-NEW.M
最后修改 : 2017/1/12 14:35:37 : asp
分析方法 : C:\CHEM32\1\METHODS\FID-肉桂酸.M
最后修改 : 2017/3/28 10:30:28 : asp
(调用后修改)
附加信息: 峰已手动积分

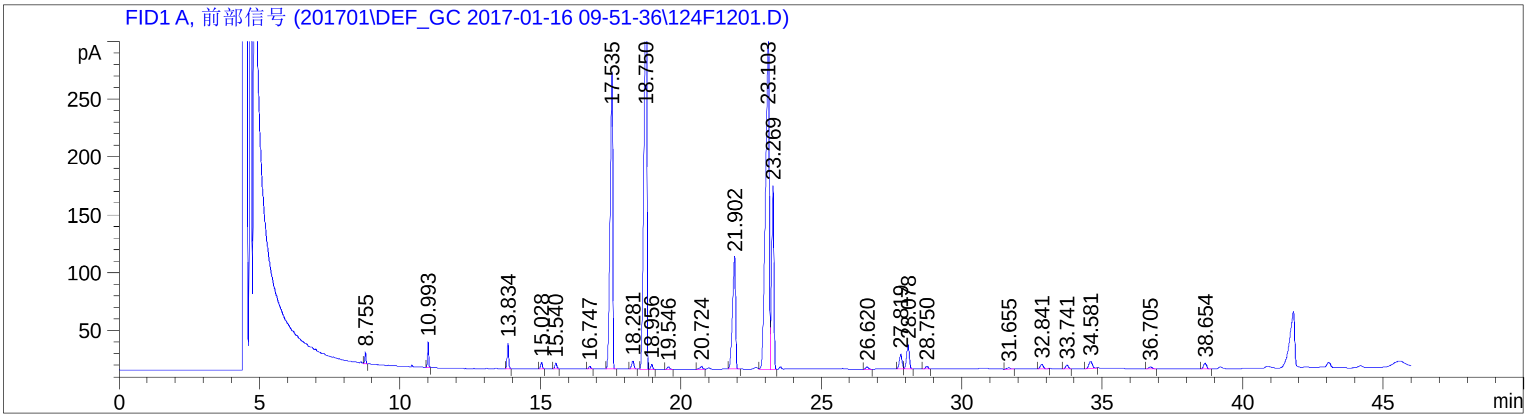

==
面积百分比报告
==

排序 : 信号
乘积因子: : 1.0000
稀释因子: : 1.0000
内标使用乘积因子和稀释因子

信号 1: FID1 A, 前部信号

峰 #	保留时间 [min]	类型	峰宽 [min]	峰面积 [pA*s]	峰高 [pA]	峰面积 %
1	8.755	BB	0.0443	25.73637	9.89622	0.27607
2	10.993	BB	0.0490	65.54993	21.85394	0.70315
3	13.834	BB	0.0620	86.41131	21.89881	0.92693
4	15.028	BB	0.0677	22.03772	5.16848	0.23640
5	15.540	BB	0.0757	25.13142	5.26609	0.26958
6	16.747	BB	0.0751	11.23218	2.37830	0.12049
7	17.535	BB	0.1030	1633.73083	254.12425	17.52501
8	18.281	BB	0.0973	42.13620	6.89073	0.45199
9	18.750	BV	0.0986	2670.17725	396.12265	28.64296
10	18.956	VB	0.0725	20.03854	4.28742	0.21495
11	19.546	BB	0.0944	13.40254	2.22256	0.14377
12	20.724	BV	0.1112	17.79153	2.32576	0.19085
13	21.902	BB	0.1167	733.13843	96.42939	7.86437
14	23.103	VV	0.1377	2638.16895	280.61423	28.29961
15	23.269	VB	0.0872	883.63269	158.19196	9.47872
16	26.620	BB	0.1025	14.14943	2.15753	0.15178
17	27.819	BV	0.1100	85.97517	12.53864	0.92225
18	28.078	VB	0.1084	140.58792	20.92276	1.50808

峰 #	保留时间 [min]	类型	峰宽 [min]	峰面积 [pA*s]	峰高 [pA]	峰面积 %
19	28.750	MM R	0.1105	17.44535	2.63184	0.18714
20	31.655	BB	0.1207	9.85541	1.29570	0.10572
21	32.841	BB	0.1197	30.61681	3.98067	0.32843
22	33.741	MM R	0.1273	26.73068	3.50021	0.28674
23	34.581	BB	0.1448	56.08152	6.01264	0.60159
24	36.705	BB	0.1290	14.76415	1.70547	0.15837
25	38.654	BB	0.1215	37.75890	5.03431	0.40504

总量 : 9322.28124 1327.45056

===

*** 报告结束 ***
