# Supplementary material for: The Transcriptional Factor PPARαb Positively Regulates Elovl5 Elongase in Golden Pompano Trachinotus ovatus (Linnaeus 1758)
Source: Front Physiol. 2018 Sep 25;9:1340. doi: 10.3389/fphys.2018.01340 (PMC6167968; doi:10.3389/fphys.2018.01340)
Supplement: Supplementary file 10 [file Data_Sheet_6.PDF]

数据文件: C:\CHEM32\1\DATA\201701\DEF_GC 2017-01-16 09-51-36\124F1301.D
样品名称: BW4482-20-2

===

操作者 : asp 序列行 : 13
仪器 : 仪器 1 位置 : 样品瓶 124
进样日期 : 2017/1/16 19:49:03 进样次数 : 1
进样量 : 1 µl
采集方法 : C:\CHEM32\1\DATA\201701\DEF_GC 2017-01-16 09-51-36\FID-脂肪酸HP88-NEW.M
最后修改 : 2017/1/12 14:35:37 : asp
分析方法 : C:\CHEM32\1\METHODS\FID-肉桂酸.M
最后修改 : 2017/3/28 10:30:28 : asp
(调用后修改)
附加信息: 峰已手动积分

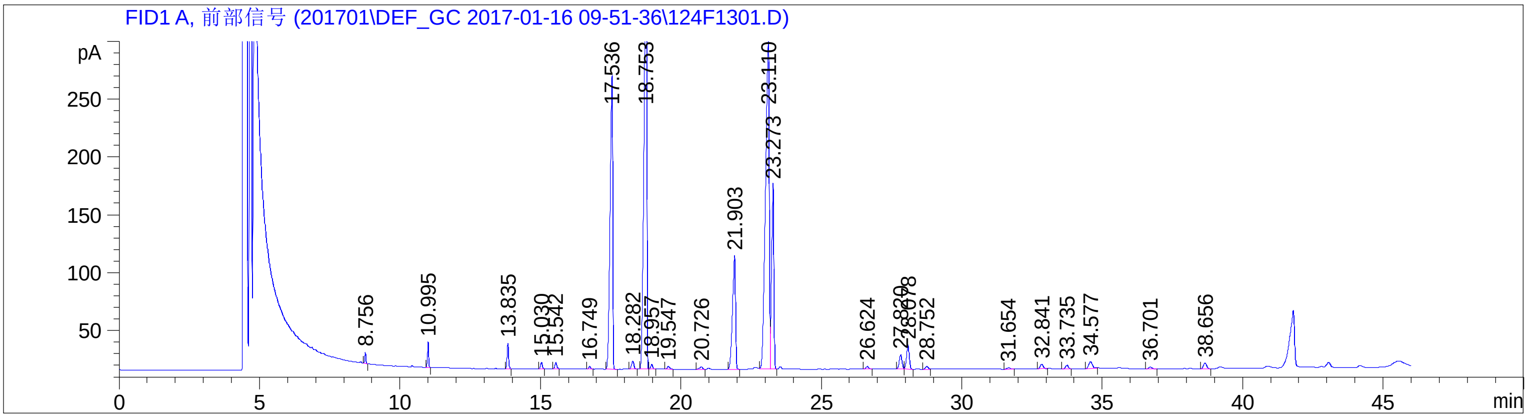

===
面积百分比报告
===

排序 : 信号
乘积因子: : 1.0000
稀释因子: : 1.0000
内标使用乘积因子和稀释因子

信号 1: FID1 A, 前部信号

峰 #	保留时间 [min]	类型	峰宽 [min]	峰面积 [pA*s]	峰高 [pA]	峰面积 %
1	8.756	BB	0.0423	25.58344	9.86312	0.27571
2	10.995	BB	0.0468	65.23398	21.84908	0.70301
3	13.835	BB	0.0619	85.87666	21.83018	0.92548
4	15.030	BB	0.0671	22.01249	5.22742	0.23722
5	15.542	BB	0.0761	25.39119	5.27929	0.27364
6	16.749	BB	0.0743	11.03295	2.36888	0.11890
7	17.536	BB	0.1050	1624.84277	252.69876	17.51062
8	18.282	BB	0.0983	41.18065	6.64606	0.44380
9	18.753	BV	0.1063	2657.17871	395.75311	28.63591
10	18.957	VB	0.0768	19.67970	4.19010	0.21208
11	19.547	BB	0.0947	13.41383	2.21210	0.14456
12	20.726	BV	0.1120	17.00919	2.25401	0.18330
13	21.903	BB	0.1169	728.84003	97.82008	7.85457
14	23.110	FM R	0.1531	2631.35767	286.42026	28.35765
15	23.273	VV	0.0907	881.15833	158.83543	9.49608
16	26.624	BB	0.1070	14.41720	2.12795	0.15537
17	27.820	BV	0.1122	85.22651	12.09662	0.91847
18	28.078	VB	0.1043	139.84796	20.84426	1.50712

峰 #	保留时间 [min]	类型	峰宽 [min]	峰面积 [pA*s]	峰高 [pA]	峰面积 %
19	28.752	MM R	0.1229	20.87684	2.83138	0.22499
20	31.654	BB	0.1199	9.77588	1.29812	0.10535
21	32.841	BB	0.1114	26.32065	3.77138	0.28365
22	33.735	MM R	0.1270	25.39851	3.33360	0.27371
23	34.577	BB	0.1476	56.18842	5.76911	0.60553
24	36.701	BB	0.1377	13.65701	1.59851	0.14718
25	38.656	BB	0.1175	37.68167	5.02180	0.40609

总量 : 9279.18223 1331.94062

==

*** 报告结束 ***
