# Supplementary material for: The Transcriptional Factor PPARαb Positively Regulates Elovl5 Elongase in Golden Pompano Trachinotus ovatus (Linnaeus 1758)
Source: Front Physiol. 2018 Sep 25;9:1340. doi: 10.3389/fphys.2018.01340 (PMC6167968; doi:10.3389/fphys.2018.01340)
Supplement: Supplementary file 11 [file Data_Sheet_7.PDF]

数据文件: C:\CHEM32\1\DATA\201701\DEF_GC 2017-01-16 09-51-36\125F1401.D
样品名称: BW4482-21-1

===

操作者	: asp	序列行 :	14
仪器	: 仪器 1	位置 :	样品瓶 125
进样日期	: 2017/1/16 20:38:32	进样次数 :	1
		进样量 :	1 µl

采集方法 : C:\CHEM32\1\DATA\201701\DEF_GC 2017-01-16 09-51-36\FID-脂肪酸HP88-NEW.M
最后修改 : 2017/1/12 14:35:37 : asp
分析方法 : C:\CHEM32\1\METHODS\FID-肉桂酸.M
最后修改 : 2017/3/28 10:30:28 : asp
(调用后修改)
附加信息: 峰已手动积分

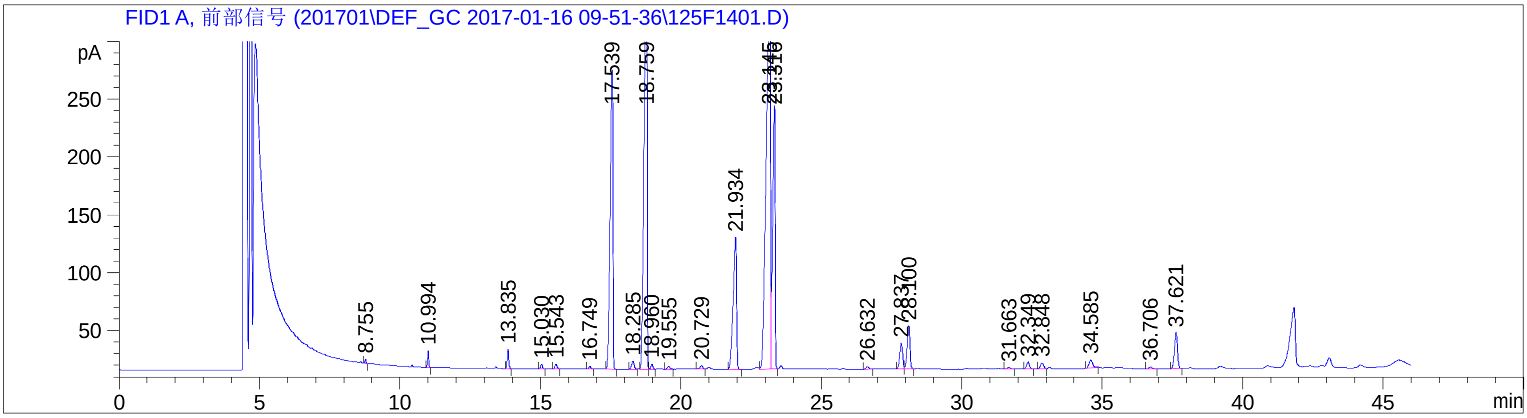

===
面积百分比报告
===

排序 : 信号
乘积因子: : 1.0000
稀释因子: : 1.0000
内标使用乘积因子和稀释因子

信号 1: FID1 A, 前部信号

峰 #	保留时间 [min]	类型	峰宽 [min]	峰面积 [pA*s]	峰高 [pA]	峰面积 %
1	8.755	BB	0.0443	9.75571	3.75470	0.08421
2	10.994	BB	0.0494	43.88643	14.48267	0.37881
3	13.835	BB	0.0651	68.82540	17.05666	0.59408
4	15.030	BB	0.0684	17.07977	3.95567	0.14743
5	15.543	BB	0.0809	21.65414	4.28850	0.18691
6	16.749	BB	0.0795	10.64052	2.15793	0.09185
7	17.539	BB	0.1030	1751.26257	258.74429	15.11627
8	18.285	BB	0.0984	44.26365	6.94206	0.38207
9	18.759	BV	0.1024	2895.15796	419.95389	24.98997
10	18.960	VB	0.0744	20.97231	4.49493	0.18103
11	19.555	BB	0.1012	15.34837	2.32056	0.13248
12	20.729	BV	0.1174	22.98614	2.87045	0.19841
13	21.934	BB	0.1261	957.65424	113.83385	8.26613
14	23.145	VV	0.1509	3523.39868	323.90863	30.41272
15	23.316	VV	0.0913	1343.49109	226.15709	11.59653
16	26.632	BB	0.1111	14.28891	2.05552	0.12334
17	27.837	BV	0.1142	159.77130	22.14376	1.37909
18	28.100	VB	0.1078	259.81833	37.07468	2.24266

峰 #	保留时间 [min]	类型	峰宽 [min]	峰面积 [pA*s]	峰高 [pA]	峰面积 %
19	31.663	BB	0.1241	9.78870	1.21399	0.08449
20	32.349	BB	0.1131	41.49390	5.82357	0.35816
21	32.848	BV	0.1202	42.82408	5.41914	0.36964
22	34.585	BB	0.1479	68.76764	6.92119	0.59358
23	36.706	BB	0.1437	13.43327	1.45566	0.11595
24	37.621	BB	0.1184	228.71791	30.88700	1.97421

总量 : 1.15853e4 1517.91640

*** 报告结束 ***
