# Supplementary material for: The Transcriptional Factor PPARαb Positively Regulates Elovl5 Elongase in Golden Pompano Trachinotus ovatus (Linnaeus 1758)
Source: Front Physiol. 2018 Sep 25;9:1340. doi: 10.3389/fphys.2018.01340 (PMC6167968; doi:10.3389/fphys.2018.01340)
Supplement: Supplementary file 12 [file Data_Sheet_8.PDF]

数据文件: C:\CHEM32\1\DATA\201701\DEF_GC 2017-01-16 09-51-36\125F1501.D
样品名称: BW4482-21-2

===

操作者 : asp 序列行 : 15
仪器 : 仪器 1 位置 : 样品瓶 125
进样日期 : 2017/1/16 21:28:08 进样次数 : 1
进样量 : 1 µl
采集方法 : C:\CHEM32\1\DATA\201701\DEF_GC 2017-01-16 09-51-36\FID-脂肪酸HP88-NEW.M
最后修改 : 2017/1/12 14:35:37 : asp
分析方法 : C:\CHEM32\1\METHODS\FID-肉桂酸.M
最后修改 : 2017/3/28 10:30:28 : asp
(调用后修改)
附加信息: 峰已手动积分

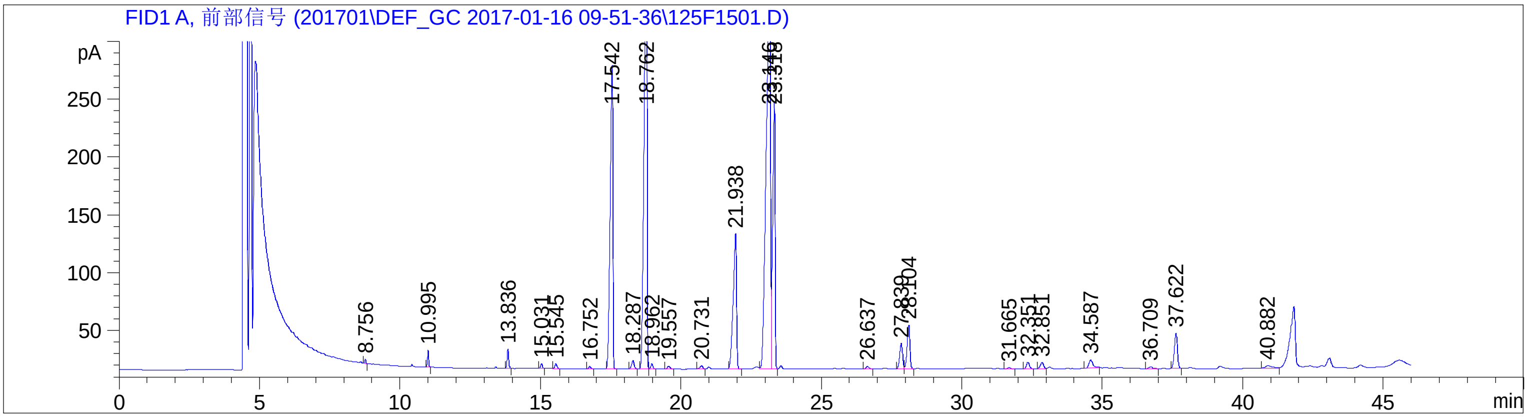

===
面积百分比报告
===

排序 : 信号
乘积因子: : 1.0000
稀释因子: : 1.0000
内标使用乘积因子和稀释因子

信号 1: FID1 A, 前部信号

峰 #	保留时间 [min]	类型	峰宽 [min]	峰面积 [pA*s]	峰高 [pA]	峰面积 %
1	8.756	BB	0.0430	9.97993	3.75204	0.08574
2	10.995	BB	0.0473	43.99058	14.53918	0.37792
3	13.836	BB	0.0656	68.93931	16.90199	0.59225
4	15.031	BB	0.0681	17.27599	4.02649	0.14842
5	15.545	BB	0.0807	21.68014	4.31043	0.18625
6	16.752	BB	0.0791	10.54547	2.15646	0.09060
7	17.542	BB	0.1000	1751.17212	261.83447	15.04422
8	18.287	BB	0.0988	44.91184	7.00246	0.38584
9	18.762	BV	0.0961	2898.82300	432.86078	24.90362
10	18.962	VB	0.0747	21.10619	4.50047	0.18132
11	19.557	BB	0.1060	15.40545	2.30415	0.13235
12	20.731	BV	0.1144	21.99164	2.83962	0.18893
13	21.938	BB	0.1180	959.25775	116.56542	8.24093
14	23.146	VV	0.1443	3526.54175	325.78107	30.29632
15	23.318	VV	0.0942	1344.95227	230.06783	11.55441
16	26.637	BB	0.1088	14.55463	2.10128	0.12504
17	27.839	BV	0.1135	159.09048	21.71128	1.36674
18	28.104	VB	0.1085	259.51312	37.61819	2.22946

数据文件: C:\CHEM32\1\DATA\201701\DEF_GC 2017-01-16 09-51-36\125F1501.D
样品名称: BW4482-21-2

峰 #	保留时间 [min]	类型	峰宽 [min]	峰面积 [pA*s]	峰高 [pA]	峰面积 %
19	31.665	BB	0.1251	9.62281	1.20576	0.08267
20	32.351	BB	0.1157	41.65301	5.66721	0.35784
21	32.851	BV	0.1223	43.39300	5.48755	0.37279
22	34.587	BB	0.1714	74.31509	6.91249	0.63844
23	36.709	BB	0.1480	13.46030	1.48135	0.11564
24	37.622	BB	0.1169	228.44449	30.66928	1.96255
25	40.882	MM R	0.2997	39.54610	2.19948	0.33974

总量 : 1.16402e4 1544.49672

===

*** 报告结束 ***
