# Supplementary material for: The Transcriptional Factor PPARαb Positively Regulates Elovl5 Elongase in Golden Pompano Trachinotus ovatus (Linnaeus 1758)
Source: Front Physiol. 2018 Sep 25;9:1340. doi: 10.3389/fphys.2018.01340 (PMC6167968; doi:10.3389/fphys.2018.01340)
Supplement: Supplementary file 13 [file Data_Sheet_9.PDF]

数据文件: C:\CHEM32\1\DATA\201701\DEF_GC 2017-01-16 09-51-36\126F1601.D
样品名称: BW4482-22-1

===

操作者	: asp	序列行 :	16
仪器	: 仪器 1	位置 :	样品瓶 126
进样日期	: 2017/1/16 22:17:56	进样次数 :	1
		进样量 :	1 µl

采集方法 : C:\CHEM32\1\DATA\201701\DEF_GC 2017-01-16 09-51-36\FID-脂肪酸HP88-NEW.M
最后修改 : 2017/1/12 14:35:37 : asp
分析方法 : C:\CHEM32\1\METHODS\FID-肉桂酸.M
最后修改 : 2017/3/28 10:30:28 : asp
(调用后修改)
附加信息: 峰已手动积分

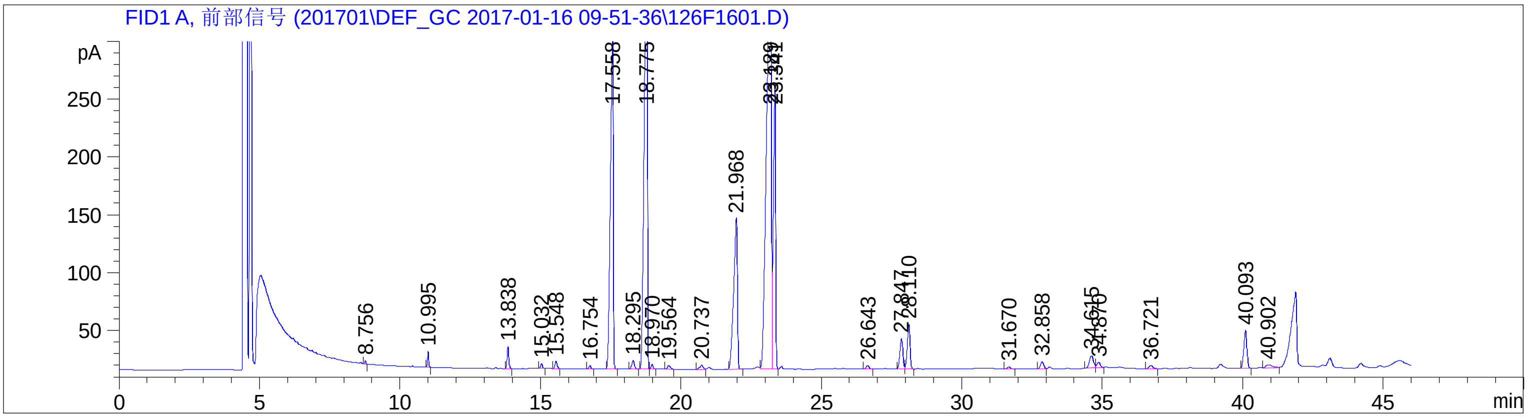

===
面积百分比报告
===

排序 : 信号
乘积因子: : 1.0000
稀释因子: : 1.0000
内标使用乘积因子和稀释因子

信号 1: FID1 A, 前部信号

峰 #	保留时间 [min]	类型	峰宽 [min]	峰面积 [pA*s]	峰高 [pA]	峰面积 %
1	8.756	BB	0.0424	8.37162	3.21326	0.06108
2	10.995	BB	0.0497	40.73366	13.31831	0.29720
3	13.838	BB	0.0674	81.80814	19.33355	0.59689
4	15.032	BB	0.0696	18.13445	4.09930	0.13231
5	15.548	BB	0.0820	33.20370	6.24718	0.24226
6	16.754	BB	0.0788	14.05320	2.78775	0.10253
7	17.558	BB	0.1087	2079.30103	293.37811	15.17094
8	18.295	BB	0.1058	47.99046	7.19610	0.35015
9	18.775	BV	0.1035	3327.57764	465.01334	24.27859
10	18.970	VB	0.0739	18.56500	4.01481	0.13545
11	19.564	BB	0.1089	22.53962	3.25183	0.16445
12	20.737	BV	0.1199	29.33442	3.56966	0.21403
13	21.968	BB	0.1333	1195.43506	129.93794	8.72210
14	23.189	FM R	0.1928	4401.97998	380.46939	32.11762
15	23.341	VV	0.0904	1378.20911	242.29396	10.05566
16	26.643	BB	0.1138	20.32543	2.83075	0.14830
17	27.847	BV	0.1131	190.06877	26.06499	1.38678
18	28.110	VB	0.1056	262.38034	38.46302	1.91437

数据文件: C:\CHEM32\1\DATA\201701\DEF_GC 2017-01-16 09-51-36\126F1601.D
样品名称: BW4482-22-1

峰 #	保留时间 [min]	类型	峰宽 [min]	峰面积 [pA*s]	峰高 [pA]	峰面积 %
19	31.670	BB	0.1262	14.28158	1.76799	0.10420
20	32.858	BV	0.1261	51.57630	6.39573	0.37631
21	34.615	BV	0.1595	109.18353	10.66237	0.79662
22	34.870	VB	0.1264	39.18181	4.74122	0.28588
23	36.721	BB	0.1432	25.23129	2.74714	0.18409
24	40.093	BB	0.1192	248.13516	32.43647	1.81044
25	40.902	MM R	0.2890	48.20964	2.78057	0.35175

总量 : 1.37058e4 1707.01475

===

*** 报告结束 ***
