# Supplementary material for: The Transcriptional Factor PPARαb Positively Regulates Elovl5 Elongase in Golden Pompano Trachinotus ovatus (Linnaeus 1758)
Source: Front Physiol. 2018 Sep 25;9:1340. doi: 10.3389/fphys.2018.01340 (PMC6167968; doi:10.3389/fphys.2018.01340)
Supplement: Supplementary file 14 [file Data_Sheet_10.PDF]

数据文件: C:\CHEM32\1\DATA\201701\DEF_GC 2017-01-16 09-51-36\126F1701.D
样品名称: BW4482-22-2

===

操作者	: asp	序列行 :	17
仪器	: 仪器 1	位置 :	样品瓶 126
进样日期	: 2017/1/16 23:07:39	进样次数 :	1
		进样量 :	1 µl

采集方法 : C:\CHEM32\1\DATA\201701\DEF_GC 2017-01-16 09-51-36\FID-脂肪酸HP88-NEW.M
最后修改 : 2017/1/12 14:35:37 : asp
分析方法 : C:\CHEM32\1\METHODS\FID-肉桂酸.M
最后修改 : 2017/3/28 10:30:28 : asp
(调用后修改)
附加信息: 峰已手动积分

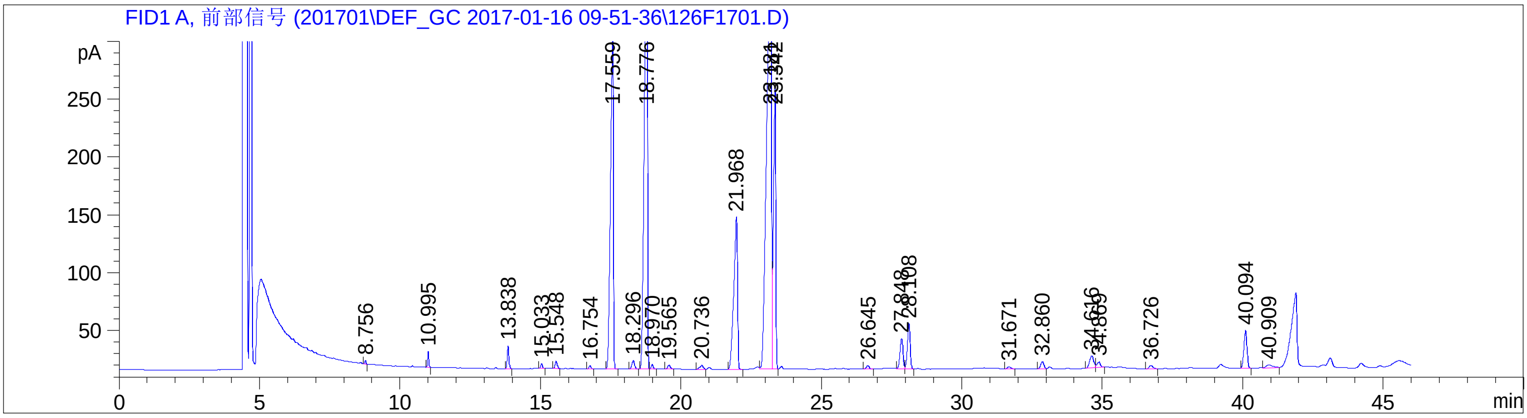

===
面积百分比报告
===

排序 : 信号
乘积因子: : 1.0000
稀释因子: : 1.0000
内标使用乘积因子和稀释因子

信号 1: FID1 A, 前部信号

峰 #	保留时间 [min]	类型	峰宽 [min]	峰面积 [pA*s]	峰高 [pA]	峰面积 %
1	8.756	BB	0.0423	8.34533	3.21582	0.06051
2	10.995	BB	0.0495	40.96064	13.45649	0.29699
3	13.838	BB	0.0649	82.21191	19.61884	0.59609
4	15.033	BB	0.0697	18.26158	4.12295	0.13241
5	15.548	BB	0.0855	33.30351	6.12018	0.24147
6	16.754	BB	0.0807	14.11330	2.80423	0.10233
7	17.559	BB	0.1151	2091.48706	293.50708	15.16469
8	18.296	BB	0.1066	48.15717	7.14387	0.34917
9	18.776	BV	0.1123	3347.46924	463.17715	24.27141
10	18.970	VB	0.0758	18.51694	4.01255	0.13426
11	19.565	BB	0.1102	22.74255	3.22789	0.16490
12	20.736	BV	0.1211	28.77340	3.45846	0.20863
13	21.968	BB	0.1310	1202.99670	131.13110	8.72254
14	23.181	FM R	0.1951	4438.17188	379.07355	32.17974
15	23.342	VV	0.0930	1381.82227	240.70233	10.01915
16	26.645	BB	0.1139	20.53193	2.78956	0.14887
17	27.848	BV	0.1189	192.67729	25.86686	1.39704
18	28.108	VB	0.1051	264.96933	39.07335	1.92121

峰 #	保留时间 [min]	类型	峰宽 [min]	峰面积 [pA*s]	峰高 [pA]	峰面积 %
19	31.671	BB	0.1238	14.57665	1.85282	0.10569
20	32.860	BV	0.1283	51.83163	6.41163	0.37581
21	34.616	BV	0.1607	108.40415	10.48220	0.78600
22	34.869	VB	0.1249	39.29443	4.82951	0.28491
23	36.726	BB	0.1421	25.38321	2.74027	0.18405
24	40.094	BB	0.1214	249.40199	32.52900	1.80833
25	40.909	MM R	0.2877	47.41410	2.74697	0.34378

总量 : 1.37918e4 1704.09468

===

*** 报告结束 ***
