# Supplementary material for: The Transcriptional Factor PPARαb Positively Regulates Elovl5 Elongase in Golden Pompano Trachinotus ovatus (Linnaeus 1758)
Source: Front Physiol. 2018 Sep 25;9:1340. doi: 10.3389/fphys.2018.01340 (PMC6167968; doi:10.3389/fphys.2018.01340)
Supplement: Supplementary file 16 [file Data_Sheet_12.PDF]

	1	2	3	4	5	6	7	8	9
C10:0	0.41	—	0.13	0.37	0.19	0.21	0.072	—	0.45
C12:0	0.59	0.36	0.66	0.87	0.65	0.57	0.31	0.2	0.64
C14:0	0.67	0.65	0.77	1.0	0.79	0.76	0.52	0.50	0.60
C14:1	0.18	0.16	0.18	0.26	0.20	0.16	0.099	0.093	0.13
C15:0	0.28	0.52	0.36	0.41	0.36	0.32	0.27	0.35	0.20
C15:1	0.13	0.21	0.13	0.19	0.16	0.12	0.12	0.14	0.10
C16:0	21.3	19.3	21.1	20.6	20.4	23.1	19.1	18.2	21.5
C16:1	35.3	33.3	28.3	36.8	36.8	34.6	32.6	31.6	34.6
C17:0	0.13	0.24	0.16	0.15	0.19	0.19	0.21	0.22	0.16
C17:1	0.18	0.23	0.15	0.21	0.24	0.23	0.23	0.26	0.20
C18:0	8.0	7.6	7.9	7.6	7.0	9.6	10.3	10.0	8.9
C18:1	26.2	27.7	20.4	27.2	25.3	28.6	30.0	35.3	27.2
C18:2	2.4	—	0.18?	0.18?	—	—	—	—	—
C18:3	—	7.2	17.0	—	—	—	—	—	—
C20:0	0.14	0.14	—	0.14	0.11	0.19	0.24	0.18	—
C20:1	—	—	—	0.055	—	0.072	—	0.095	—
C20:2	—	—	—	—	—	—	—	—	—
C20:3	—	0.087?	—	—	4.3	—	—	—	—
C20:4(ARA)	—	—	0.14?	1?	0.096?	—	3.4	—	—
C22:0	—	—	—	0.11	0.17	—	—	—	—
C22:1	0.45	0.29	0.31	0.18	—	0.73	0.44	0.28	0.42
C22:2	3.7?	—	—	—	—	0.41?	—	—	—
C23:0	—	1.7	1.8	2.2	2.7	—	—	—	—
C20:5	—	—	—	—	—	—	—	2.3	—
C24:0	—	0.13	—	0.15	0.10	—	—	0.23	—
C22:4	—	—	—	—	—	—	—	—	4.9
C24:1	—	0.059	0.11	0.10	0.084	—	—	—	—
C22:5	—	—	—	—	—	—	—	—	—
C22:6	—	—	—	—	—	—	—	—	—

10	11	12	13	14	15	16	17	18	19	20
0.58	—	0.30	0.19	0.80	0.17	0.65	0.74	0.31	0.083	0.28
0.73	0.23	0.55	0.50	0.85	0.46	0.70	1.1	0.69	0.40	0.70
0.66	0.46	0.79	0.63	0.79	0.62	0.70	0.88	0.77	0.64	0.93
0.14	—	0.17	0.14	0.19	0.15	0.17	0.26	0.17	0.14	0.24
0.20	0.31	0.39	0.29	—	0.28	0.17	0.12	0.28	0.25	0.27
0.10	—	—	—	—	0.11	0.10	0.10	0.10	0.10	0.12
19.1	19.8	22.0	19.9	20.7	19.2	16.6	19.4	18.0	15.0	17.6
32.3	30.5	25.2	29.6	34.5	32.0	32.5	29.9	22.3	23.5	29.4
—	—	—	—	—	—	0.089	—	0.16	0.18	0.14
0.11	—	—	—	—	—	0.12	—	0.13	0.26	0.19
7.2	10.4	9.1	9.0	7.6	27.8	6.1	7.2	8.6	8.8	7.9
24.0	29.6	21.5	25.8	25.6	—	23.6	34.1	28.3	39.7	37.9
—	1.8	—	—	—	—	—	2.3	0.17	—	—
—	—	12.9	—	—	—	—	—	8.2	1.4	—
—	—	—	—	—	—	0.11	2.0	0.14	0.14	0.15
—	—	—	—	—	—	—	0.63	1.7	3.6	2.4
—	—	—	—	—	—	—	—	—	—	—
—	—	—	4.0	—	—	—	—	—	2.9	—
—	—	—	—	—	—	—	—	8.1?	—	—
—	—	—	—	—	—	—	—	—	—	0.11
0.73	—	—	—	—	—	0.61	0.55	0.30	0.49	0.30
—	—	—	—	—	—	0.45?	0.51?	0.55?	0.66?	0.6?
—	6.8	7.0	9.9	9.0	8.1	—	—	—	—	—
—	—	—	—	—	—	—	—	—	—	—
—	—	—	—	—	—	—	—	0.69	0.14	0.15
—	—	—	—	—	3.0	—	—	—	—	—
—	—	—	—	—	—	—	—	—	—	—
14.0	—	—	—	—	—	17.1	—	—	—	0.41?
—	—	—	—	—	—	0.14	0.25?	0.25?	0.27?	0.24?

21	22
0.085	0.061
0.38	0.30
0.59	0.60
0.15	0.13
0.19	0.24
0.091	0.10
15.1	15.2
25.5	24.7
0.13	0.16
0.19	0.21
8.2	8.7
41.9	42.2
—	—
—	—
0.12	0.15
3.6	3.3
—	—
—	—
0.36	—
0.083	0.1
0.37	0.38
0.61?	0.79?
—	0.28
—	—?
0.12	0.18
2.0	—
—	—
—	1.8
0.28?	0.35?
